# One-step generation of semi-cloned zebrafish carrying a defined genetic modification

**DOI:** 10.1038/s41422-026-01266-0

**Published:** 2026-06-12

**Authors:** Yirui Ai, Shifeng Li, Siqi Liu, Jia Xu, Yunbin Zhang, Siyu Zhou, Zhou Zhou, Jinsong Li, Yiping Li

**Affiliations:** 1 Key Laboratory of Multi-Cell Systems, Shanghai Key Laboratory of Molecular Andrology, CAS Center for Excellence in Molecular Cell Science, Shanghai Institute of Biochemistry and Cell Biology, University of Chinese Academy of Sciences, Chinese Academy of Sciences, Shanghai, China; 2 Optogenetics & Synthetic Biology Interdisciplinary Research Center, State Key Laboratory of Bioreactor Engineering, East China University of Science and Technology, Shanghai, China; 3 Shanghai General Hospital, School of Medicine, Shanghai Jiao Tong University, Shanghai, China

**Keywords:** Biological techniques, Cell biology

Dear Editor,

Haploid embryonic stem cells (haESCs) from multiple species, including medaka fish,^1^ mouse,^2,3^ rat,^4^ cattle, sheep,^5^ and human,^6^ can “fertilize” oocytes to produce semi-cloned embryos/animals and thus have potential applications in generating animal models with complex modifications. In mice, androgenetic haESCs (AG-haESCs) carrying both *H19*-DMR and IG-DMR deletions can efficiently support the generation of semi-cloned pups upon intracytoplasmic AG-haESC injection (ICAHCI) into oocytes, at rates of up to 30.0% of transferred embryos. Therefore, mouse DKO-AG-haESCs have been used as a genetically tractable fertilization tool for complex genetic analyses at the organismal level.^7^ This approach, however, has remained unavailable in zebrafish, a key model organism for studying vertebrate development and diseases.

To generate AG or parthenogenetic (PG) haploid embryos, eggs or sperm were inactivated by ultraviolet (UV) irradiation and then subjected to in vitro fertilization with untreated sperm or eggs, respectively (Supplementary information, Fig. S1a). AG and PG haploid embryos successfully developed to the blastula stage in egg water at 28.5 °C. We then isolated AG or PG blastula cells for ESC derivation. Our previous attempts resulted in stable haploid cell lines from PG embryos; however, these cells were not ESCs but heart-related cell lines.^8^ Moreover, the reconstructed embryos generated by injection of these cells into oocytes failed to develop to the larval stage (data not shown).

Given that ESCs are derived from the inner cell mass of blastocysts, we next asked whether haploid cells from blastulas could support the ontogeny of zebrafish in a manner similar to sperm. To this end, we adopted a conventional procedure used for nuclear transfer (NT) in zebrafish and medaka fish^9^ (Supplementary information, Video S1). Using intracytoplasmic haploid blastula cell injection (ICHBCI), we found that both AG and PG haploid blastula cells could successfully support the development of reconstructed embryos to the blastula stage. However, the majority of reconstructed embryos exhibited developmental arrest and morphological abnormalities, beginning at the blastula stage (Supplementary information, Table S1). Moreover, ~63.0% of the reconstructed embryos were parthenogenetic because they did not show donor-cell fluorescence at 1 day post reconstruction (dpr). The semi-cloned embryos expressed GFP from the donor and mCherry from the recipient at 3 dpr (Fig. 1a). In this preliminary experiment, we obtained 3 adult zebrafish from 931 embryos reconstructed using AG or PG haploid blastula cells (Fig. 1b; Supplementary information, Table S1). Ploidy analysis indicated that two zebrafish ( ~0.2% of reconstructed embryos) were diploid (Fig. 1c). These two semi-cloned zebrafish were female and could produce normal offspring that carried genetic traits from the founders (Fig. 1d; Supplementary information, Fig. S2). Together, these findings demonstrate that haploid blastula cells, when injected into oocytes, can give rise to fertile semi-cloned zebrafish.

**Fig. 1:**
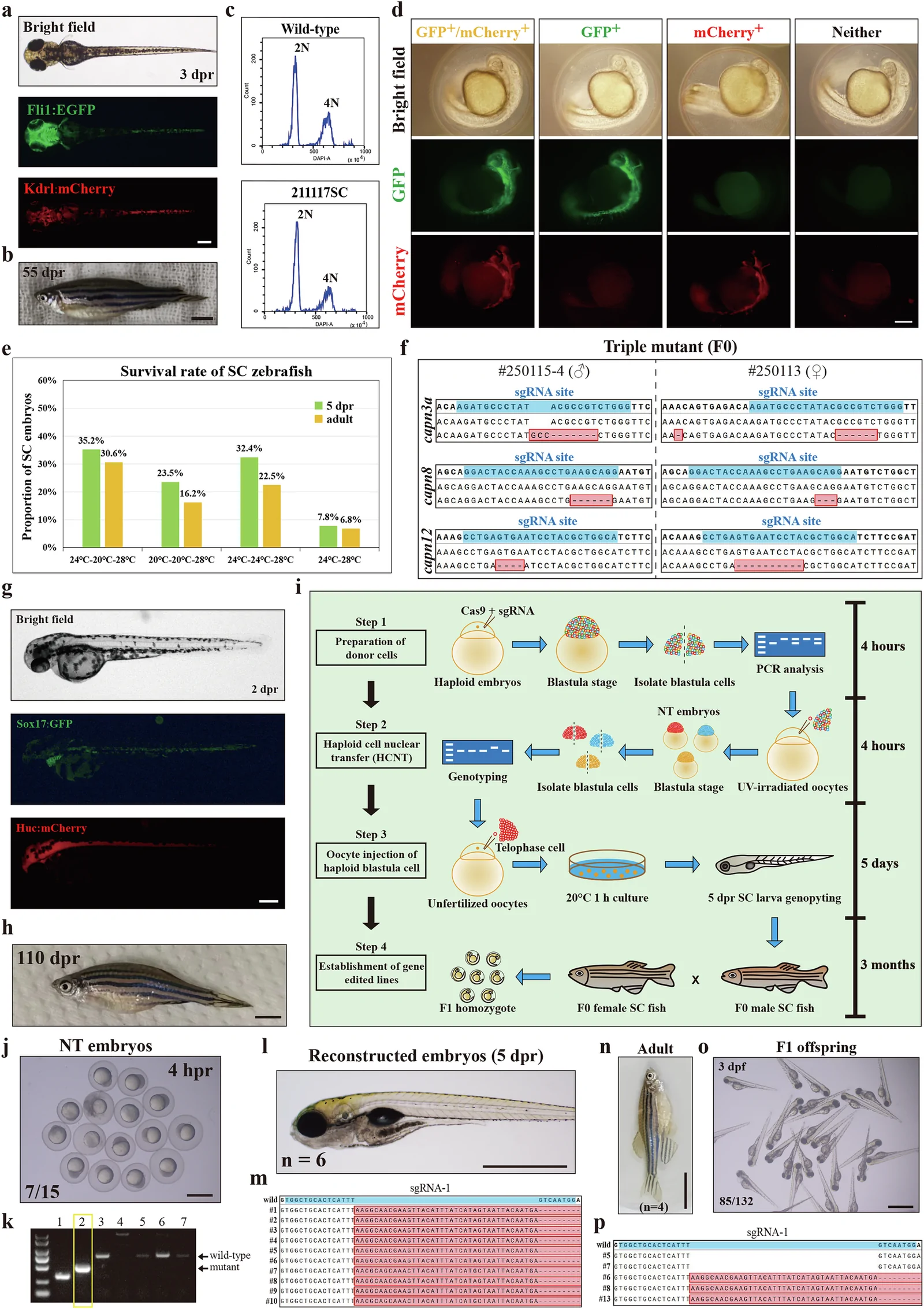
Generation of semi-cloned zebrafish carrying genetic modifications via ICHBCI. **a** Dual fluorescence images of 211117SC zebrafish (3 dpr) showing GFP expression from donor-derived Tg(*fli1a:EGFP*) zebrafish and mCherry expression from recipient Tg(*kdrl:mCherry*) zebrafish (scale bar, 250 μm). **b** Adult 211117SC zebrafish at 55 dpr following tail clipping and sampling for ploidy analysis and genotyping (scale bar, 5 mm). **c** FACS ploidy analysis showing comparable DNA content profiles between wild-type fish and 211117SC zebrafish, both exhibiting diploid (2 N) and tetraploid (4 N) peaks. **d** Fluorescence-based classification of F1 progeny into four phenotypic groups according to inheritance patterns. Dual-fluorescent embryos carried both transgenes, while non-fluorescent individuals lacked both reporters. The phenotypic ratio among the F1 offspring was 65:63:80:69 for the four types (scale bar, 200 μm). **e** Assessment of embryo (5 dpr) and adult survival rates of semi-cloned zebrafish using telophase donor cells under four thermal programs. **f** Genotyping results showing that semi-cloned zebrafish #250115-4 harbored a 4-bp indel in *capn3a*, a 6-bp deletion in *capn8*, and a 4-bp deletion in *capn12*. Semi-cloned zebrafish #250113 harbored a 7-bp deletion in *capn3a*, a 3-bp deletion in *capn8*, and a 10-bp deletion in *capn12*. **g** Morphology of zebrafish 240425SC-1 at 2 dpr. Green fluorescence (Sox17:GFP) confirms donor-derived cells, while red fluorescence (Huc:mCherry) indicates successful knock-in (scale bar, 250 μm). **h** Adult zebrafish 240425SC-1 at 110 dpr (scale bar, 5.0 mm). **i** Schematic of the NT-SC workflow: (1) RNPs were microinjected into 1-cell-stage haploid PG embryos (50 RNP-injected embryos). One half of each blastula was subjected to PCR; the contralateral half served as the nuclear donor. (2) Donor cells were microinjected into UV-inactivated, enucleated oocytes to generate reconstructed NT embryos (one embryo per haploid donor cell; 20 NT embryos). (3) A genotyped blastula was used to generate semi-cloned embryos (100 reconstructed embryos). (4) Intercrosses of F0 adults led to F1 offspring homozygous for the mutations. **j** Bright-field images of NT embryo development (scale bar, 1.0 mm). hpr hour post reconstruction. **k** PCR genotyping of NT blastulas confirming allele transmission. **l** Bright-field images of NT-SC embryo morphology (5 dpr; scale bar, 1.0 mm). **m** Mutant allele sequencing: identical deletion profiles across semi-cloned embryos confirmed clonal genetic fidelity. **n** Adult NT-SC zebrafish morphology (2 months old; scale bar, 1.0 cm). **o** F1 offspring morphology (scale bar, 1.0 mm). **p** Sequencing confirming the same deletion in F1 offspring.

To improve the success rate of semi-cloned zebrafish generation, we performed several optimization experiments. Since preparation of PG haploid embryos is easier and more efficient than that of AG haploid embryos (Supplementary information, Fig. S1b–e), we selected PG haploid embryos as the donor cell source in subsequent experiments. Next, we used a piezo-impact pipette drive unit, a standard system for mouse cloning and semi-cloning studies, to replace the conventional pneumatic operation system used in zebrafish embryo manipulation, to greatly reduce trauma (Supplementary information, Video S2). Surprisingly, 23.4% of the reconstructed embryos developed to 1 dpr, and approximately 60.0% of them carried the donor genome (Supplementary information, Table S1). Importantly, seven adult zebrafish were obtained, including five diploid semi-cloned individuals (approximately 3.0% of reconstructed embryos) and two triploid zebrafish (Supplementary information, Fig. S3). All tested semi-cloned fish were fertile.

In this series of experiments, however, we still observed that ~40.0% of the reconstructed embryos developed parthenogenetically. Given that haploid donor cells in M phase are critical for the development of reconstructed semi-cloned embryos in mice,^3^ we treated blastula cells with nocodazole to synchronize them in M phase (Supplementary information, Fig. S4). Strikingly, over 90.0% of reconstructed embryos (27 of 29, 1 dpr) carried the genome from the injected cell (Supplementary information, Fig. S5a); however, 9 of 10 reconstructed embryos at 5 dpr were triploid (Supplementary information, Fig. S5b, c). We speculated that M phase haploid cells do not have a sufficient time window for exclusion of the pseudo-polar body, thus forming a diploid pseudo-pronucleus and a triploid karyotype in the reconstructed embryos. To address this problem, we attempted to use telophase haploid donor cells, which contain one set of chromosomes at the final stage of mitosis, for injection (Supplementary information, Fig. S6a and Video S3). Usually, the two daughter cells that remain attached during late cytokinesis are in telophase. We thus picked one of the two attached cells for injection. Strikingly, ~80.0% of reconstructed embryos (21 of 26, 1 dpr) were diploid (Supplementary information, Fig. S6b–d).

However, the overall developmental potential of semi-cloned embryos was still low, probably due to asynchrony between the pseudo-pronucleus and female pronucleus caused by rapid proliferation in zebrafish embryos. We thus treated the semi-cloned embryos at a low temperature, which can slow the growth rate of zebrafish embryos without compromising their developmental potential.^10^ We tested different temperature conditions and found that exposure to 20 °C for 1 h before long-term culture under normal conditions dramatically enhanced the developmental potential of reconstructed embryos. Remarkably, 35.2% of reconstructed embryos derived from freshly isolated telophase blastula cells developed normally to 5 dpr; of these, 86.8% (33 of 38) further developed into adults with diploid karyotypes (Fig. 1e; Supplementary information, Table S2). Collectively, these refinements increased the overall efficiency of deriving adult semi-cloned zebrafish to 30.6%, 150-fold higher than that of the initial method.

Interestingly, PG haploid blastula cells gave rise to both male and female semi-cloned zebrafish (Supplementary information, Table S3). This phenomenon is different from that observed in semi-cloned animals in other species, including medaka fish, mice, and rats, all of which are female,^1,3,4^ probably due to the unique sex determination mechanisms and absence of imprinting in zebrafish.^11^ Moreover, both female and male semi-cloned zebrafish were fertile. The female-to-male ratio was approximately 2.12:1 (Supplementary information, Table S4), probably due to the low rearing density in our study, a finding consistent with previous observations that doubling food availability increases the proportion of females.^11^ Taken together, these results show that we have developed a protocol for the efficient generation of both female and male semi-cloned zebrafish.

In mice, semi-cloning technology in combination with CRISPR/Cas9 has been used to efficiently generate mouse models with uniform gene modifications in one step and to avoid mosaicism in founder animals.^12^ We asked whether mutant zebrafish with a single genotype could be generated via the semi-cloning system established here. To test this, we first injected Cas9 protein and sgRNA targeting the *tbxta* gene into one-cell PG haploid embryos (Supplementary information, Fig. S7a, Strategy A). Next, when the injected embryos reached the blastula stage, single haploid cells were dissected and randomly selected for injection. From 230 reconstructed embryos, we obtained 4 adult semi-cloned zebrafish, all of which carried heterozygous *tbxta* mutations (Supplementary information, Fig. S7b). These zebrafish were fertile and successfully transmitted the mutant *tbxta* gene to the next generation (Supplementary information, Fig. S7c). As expected, all analyzed offspring carried the same genotype as the founder.

Subsequently, we investigated whether multiple genetic alterations could be introduced into haploid blastula cells by simultaneously disrupting *tbxta*, *smo*, and *tfap2a*, followed by the generation of semi-cloned zebrafish carrying multiple mutations. Using blastula-stage cells as donors, we obtained 6 adult semi-cloned zebrafish from 311 reconstructed embryos: 5 with triple-gene mutations and 1 with double-gene mutations (Supplementary information, Fig. S7d). All of these founders carried different mutations in the three targeted genes, which were transmitted to F1 offspring that retained the same genotypes as the founders (Supplementary information, Fig. S7e). We also attempted to introduce mutations in 3 genes from the same family: *capn3a*, *capn8*, and *capn12* (Supplementary information, Fig. S8a). From 71 reconstructed embryos, we generated 7 adult F0 fish with distinct mutation profiles; among these, 2 carried triple mutations (Fig. 1f). Interestingly, female and male semi-cloned founders with identical mutations in *capn3a* and *capn12* (Supplementary information, Fig. S8b) could give rise to *capn3a*/*capn12* homozygous mutant zebrafish in the F1 generation (Supplementary information, Fig. S8c). Similarly, a cross between two zebrafish carrying different mutations in all three genes led to triple biallelic mutants (Supplementary information, Fig. S8d). Together, these results demonstrate that the haploid blastula cell-mediated semi-cloning method enables the efficient generation of zebrafish with multiplex mutations, thus dramatically accelerating the derivation of multiplex mutant zebrafish.

We next investigated whether precise gene modification via the insertion of a DNA construct into an endogenous gene was feasible in haploid blastulas and could further lead to knock-in semi-cloned zebrafish. For this purpose, we first co-injected Cas9 protein, sgRNA, and a *huc*-mCherry knock-in plasmid (Supplementary information, Fig. S9a) into PG haploid embryos. Less than 2.0% of injected embryos displayed red fluorescence at 1 day post fertilization (dpf), indicating the low efficiency of generating knock-in zebrafish through zygote injection.^13^ To select chimeric blastulas with a high probability of gene knock-in as donors, we established a pre-screening protocol (Supplementary information, Fig. S9b, Strategy B), in which each haploid blastula embryo was bisected, with one half subjected to PCR analysis while the matched half was preserved at 4 °C for later injection. From 300 injected PG haploid embryos, ~90.0% developed to the blastula stage, and PCR analysis confirmed that 4 embryos were *mCherry*-positive; thus, the haploid blastula cells from the matched half were used as donors for oocyte injection (Supplementary information, Fig. S9c). Of the 83 reconstructed embryos, 4 of 11 semi-cloned embryos displayed red fluorescence at 2 dpr (Fig. 1g); of these, two developed to adulthood (Fig. 1h; Supplementary information, Fig. S9d), and these two heterozygous knock-in semi-cloned zebrafish successfully transmitted the genetic trait to F1 offspring (Supplementary information, Fig. S9e, f). Thus, semi-cloning technology combined with pre-selection of blastulas with a specific genetic trait enables the efficient generation of heterozygous knock-in zebrafish.

Although semi-cloning technology can efficiently give rise to gene-modified zebrafish, these founders carry distinct genotypes due to genotypic mosaicism in donor blastulas caused by direct injection of CRISPR-Cas9 into 1-cell haploid embryos. To generate semi-cloned zebrafish with a defined genetic deletion, we designed a nuclear transfer-semi-cloning (NT-SC) strategy (Fig. 1i, Strategy C), in which gene-modified haploid blastula cells undergo clonal expansion via nuclear transfer. To test this, we attempted to generate zebrafish with a *capn12* exon 3 deletion. Cas9 protein and two sgRNAs targeting exon 3 of *capn12* (Supplementary information, Fig. S10a) were co-injected into 1-cell PG haploid embryos of Tg(*sox17:GFP*). At the blastula stage, half of each embryo was subjected to PCR screening to identify embryos with high editing efficiency. Of the 16 haploid embryos examined, 4 showed high editing efficiency (Supplementary information, Fig. S10b). Embryo #3, with an exclusively truncated band, was then used as a donor in cloning using UV-inactivated Tg(*kdrl:mCherry*) eggs as recipients. A total of 7 cloned blastulas (NT-blastulas) were obtained from 15 reconstructed embryos (Fig. 1j). Each NT-blastula embryo was bisected for parallel processing: one half underwent PCR and sequencing analysis, while the other half was preserved in L-15 medium at 4 °C overnight. Sequencing analysis confirmed that NT-blastula #2 harbored the expected exon 3 deletion (+39 bp, –339 bp), and the remaining half was used as a donor for injection into oocytes of the AB strain (Fig. 1k; Supplementary information, Fig. S10c). Notably, preserved cells retained mitotic capacity, and telophase cells were selected as donors. Of 40 reconstructed NT-SC embryos, 12 developed to 1 dpr (Supplementary information, Fig. S10d), and 10 showed green fluorescence but not red fluorescence, indicating successful NT-SC reconstruction (Supplementary information, Fig. S10e). Six embryos developed to 5 dpr with normal morphology (Fig. 1l). We performed genotyping analysis and confirmed that all these NT-SC embryos carried a mutant allele identical to that of NT Embryo #2 (Fig. 1m). Importantly, 4 survived to adulthood, including 2 males and 2 females (Fig. 1n), all of which were diploid (Supplementary information, Fig. S10f). Intercrosses of these adults led to F1 offspring homozygous for the *capn12* exon 3 deletion (Fig. 1o, p). Collectively, these results demonstrate that the NT-SC strategy allows the efficient generation of zebrafish with a defined genotype; subsequent breeding between male and female semi-clones leads to zebrafish with a homozygous trait in the F1 generation, thus saving at least one generation compared with conventional strategies.

Cytoplasmic injection of CRISPR/Cas9 into zygotes has been widely adopted for generating gene-modified zebrafish. However, due to the rapid cleavage dynamics of early-stage zebrafish embryos, the resulting fish are usually somatic and germline chimeras.^14^ Here, using blastula cell-mediated semi-cloning technology, we have addressed all of these technical bottlenecks. By combining cryopreservation of blastomere donor cells, PCR-based donor preselection, and nuclear transfer-mediated expansion of blastula cells, our semi-cloning system provides a robust platform for genetic analyses in zebrafish. We devised three alternative strategies for diverse experimental purposes (Supplementary information, Table S7). Strategy A (Supplementary information, Fig. S7a) is technically straightforward. It uses blastulas with high chimerism ratios as donors and is applicable to protocols that efficiently generate highly chimeric embryos, such as conventional gene knockout. Strategy B (Supplementary information, Fig. S9b) incorporates an additional PCR-based pre-screening step for donor embryos. This procedure selects blastulas with the highest proportion of gene-editedcells as suitable donors. This strategy is suitable for gene knock-in experiments. Strategy C (Fig. 1i) enables the generation of semi-cloned zebrafish with predefined genotypes but requires more complex experimental manipulation. This workflow is applicable to precise gene editing applications, including site-specific point mutations. The next challenge is to establish stable zebrafish haESCs that can be used as a sperm substitute to support the derivation of semi-cloned fish. As these cultured cells would be amenable to gene modification in vitro, they may further improve the efficiency of generating genetically modified animals.

## Supplementary information

Supplementary informaiton, Video S1 Intracytoplasmic haploid blastula cell injection using pneumatic operation system

Supplementary informaiton, Video S2 Intracytoplasmic haploid blastula cell injection using piezo system

Supplementary informaiton, Video S3 Mitotic progression of synchronized cells after release

Supplementary information, Methods, Figs and Tables

## Data Availability

All data are available in the main text or supplementary information. Zebrafish lines and plasmid are available from Y.P.L. under a material transfer agreement with the Shanghai Institute of Biochemistry and Cell Biology.
