# Supplementary material for: One-step generation of semi-cloned zebrafish carrying a defined genetic modification
Source: Cell Res. 2026 Jun 12;36(9):734–8. doi: 10.1038/s41422-026-01266-0 (PMC13503951; doi:10.1038/s41422-026-01266-0)
Supplement: Supplementary file 1 [file 41422_2026_1266_MOESM4_ESM.pdf]

SUPPLEMENTARY INFORMATION

One-step generation of semi-cloned zebrafish carrying a defined genetic modification

This PDF file includes:

Methods

Supplementary Fig. S1 to S10

Supplementary Table S1 to S7

Other Supplementary Materials for this manuscript include the following:

Supplementary Videos. S1 to S3

Methods

Zebrafish strains

The wild-type AB strain and the transgenic strains Tg(*kdrl:mCherry*)[15], Tg(*fli1a:EGFP*)[16], Tg(*sox17:GFP*)[17], Tg(*mylpfa:EGFP*)[18] and Tg(*h2afva:h2afva-GFP*)[19] were sourced from the Zebrafish Core Facility of Center for Excellence in Molecular Cell Science, Chinese Academy of Sciences (CAS), China, and were maintained under standard husbandry conditions at 28.5°C. Embryonic developmental stages were determined based on morphological characteristics, as described in the references[20]. All the fish experiments were approved by the Institutional Animal Care and Use Committee of Center for Excellence in Molecular Cell Science, and were performed in compliance with institutional guidelines.

Preparation of PG zebrafish embryos

PG zebrafish embryos were generated using a modification of a previously described method[21]. Briefly, adult males were anesthetized with tricaine (the recipe for tricaine is available online in the Zebrafish Book (http://zfin.org/zf_info/zfbook/chapt10.html#wptohtml63)) and surface moisture was blotted off with paper towels. Milky semen was collected by gently massaging the abdominal region while aspirating the released fluid with a 10 μL pipette. Sperm from 3-5 males was pooled in 0.5 mL of PBS within a 1.5 mL microcentrifuge tube. Sperm suspension was then transferred to a watch glass and subjected to UV inactivation (254 nm wavelength) for 5 or 10 seconds at a distance of ~6 cm. Subsequently, the inactivated sperm was transferred to a fresh microcentrifuge tube. For egg collection, female zebrafish were similarly anesthetized and dried before gently abdominal squeezing to release eggs into Petri dishes. Fertilization was initiated by adding 50 μL of the prepared sperm suspension to the eggs, followed by activation with 1 mL egg water under gentle agitation.

Preparation of AG zebrafish embryos

Sperm was stored in PBS on ice following the established AG embryo preparation protocol[22]. Eggs from a single female were resuspended in 1 mL of PBS and subsequently transferred onto a 100 µm mesh nylon filtration apparatus. The oocytes were subjected to UV irradiation for 5 or 10 seconds using a dual-lamp system (254 nm wavelength UV source) positioned above and below the sample platform, with a fixed 6 cm distance maintained between irradiation source and the biological material. Post-irradiation treatment, the inactivated eggs were transferred to a clean culture dish, and PBS was carefully removed using a Pasteur pipette. Fertilization was initiated by introducing 50 µL of preserved sperm suspension to the oocytes, followed by activation with 1 mL of egg water. The embryos were maintained in egg water at 28.5°C for further development.

Preparation of donor cells from AG and PG embryos

Donor cells were isolated from both PG and AG embryos at the sphere stage (4 hpf). Embryos were sequentially processed through the following protocol: 1. Embryos were rinsed twice with PBS (room temperature), and dechorionated under a stereomicroscope. 2. Yolk material was gently dissected out with forceps while preserving embryonic cells. The remaining embryonic tissue was transferred to a microcentrifuge tube containing 20 µL PBS. 3. Cell suspension was either maintained on ice for short-term storage (<1 h) or stored at 4ºC for an extended period (up to 4 hours). 4. Prior to use, cells were dissociated into a single-cell suspension by repeated pipetting (10 times) using a 20 µL pipette tip.

Recipient egg collection

Sexually mature male and female zebrafish were kept in partitioned spawning chambers overnight. Following removal of the partition the next morning, spawning activity was monitored until egg

deposition was observed. The females were immediately anesthetized in 168 mg/L tricaine (MS-222). Oocytes were collected via ventral abdominal massage into a Petri dish containing Hank′s solution supplemented with 1.5% BSA and 0.1% NaCl (w/v) which served as the oocyte preservation solution. Activation was initiated by transferring oocytes into a 10 cm culture dish filled with egg water. After precisely 20 seconds of H_2O-mediated activation, the eggs were sequentially transferred into operation medium (Hank′s solution with 1.5% BSA). All intracytoplasmic microinjections were completed within a 15-minute post-activation window to ensure optimal receptivity.

Intracytoplasmic injection of haploid embryonic cells

A standardized coordinate system was established on the underside of a 60 mm Petri dish by marking perpendicular axes with a marker pen. Following inversion of the dish, 2 mL of operational buffer (Hank’s solution supplemented with 1.5% BSA) was dispensed within the demarcated central quadrant. Temperature was maintained at 24 ± 0.5°C using a precision temperature control apparatus. Concurrently, 50 μL of washing buffer (Hank’s solution containing 10% polyvinylpyrrolidone) was dropped onto an area of the peripheral region. Prior to cellular manipulation, the 10 μm microinjection needle was triple-rinsed with the prepared washing buffer. Oocyte orientation was performed by strategically positioning recipient oocytes and donor cells in designated operational zones. Morphologically intact oocytes with clearly identifiable animal poles were selected for microinjection and properly aligned relative to the injector. The microinjection consisted of three sequential steps: 1) Donor cell aspiration and membrane disruption through controlled mechanical disruption at the needle interface. 2) Needle positioning adjustment to achieve coplanar alignment with the animal pole. 3) Membrane penetration via micropylar access using Piezo-assisted pulsed drilling. Post-injection oocytes were temporarily

maintained in quadrant II during batch processing. All manipulated embryos underwent sequential incubation in Holtfreter's solution: primary stabilization at 20°C for 60 minutes, with subsequent transfer to egg water at 28.5°C.

Preparation of nuclear transfer embryo

Nuclear transfer (NT) embryos were generated using methodology analogous to the haploid cell injection protocol, with the following modification: UV-inactivated oocytes were substituted for intact oocytes. Following anesthesia of female zebrafish in MS-222, oocytes were collected into preservation solution. For subsequent processing, 5-10 oocytes were selected and activated in egg water for 20 seconds before being returned to preservation solution to prevent over-activation, followed by UV treatment (254 nm, 10 seconds) on a 100 μm nylon mesh. The UV-inactivated oocytes were then transferred to operational buffer for nuclear transfer procedures, which were performed as previously described.

Genotyping of zebrafish

For genotyping of embryos at the sphere stage (4 hpf), embryos were transferred into Leibovitz's L-15 medium in a 60 mm Petri dish. Manual dissection was performed using fine forceps to remove both the chorion and yolk sac. One half of each blastula-stage embryo was subjected to PCR genotyping using Phire Tissue Direct PCR Master Mix (Thermo, cat: F170L), while the contralateral half was cryopreserved in L-15 medium at 4°C to maintain cellular viability for potential nuclear transfer applications. For genomic DNA extraction from zebrafish embryos (5dpr) and adult, caudal fin tissue was surgically excised using micro-scissors. The collected tissue samples were immediately transferred into 20 μL of 50 mM NaOH solution and subjected to alkaline lysis through thermal cycling: incubation at 98°C for 20 min in a thermocycler. Following

lysis, the lysate was neutralized with 2 μL Tris-HCl (1 M, pH 8.0). Subsequently, 2 μL of the neutralized lysate was directly utilized as template DNA for PCR amplification using target-specific primers (Supplementary Table 7). The amplified PCR products were directionally cloned into the T-vector using pEASY-T1 Simple Cloning Kit (TransGen Biotech, CT111) according to the manufacturer′s protocol.

F1 offspring identification and characterization

Adult semi-cloned zebrafish were outcrossed with wild-type fish to generate F1 progeny, which were subsequently characterized through multiple approaches. First, embryonic morphology and fluorescence patterns (GFP/mCherry) were observed and photographed using an Olympus IX71 fluorescence microscope. Ploidy analysis was performed at 24 hpf via flow cytometry following the established method. For genotyping, we first verified fluorescent markers using PCR with *GFP* and *mCherry* primers (Supplementary information, Table S5). Additionally, mutations in the *tbxta*, *smo*, and *tfap2a* genes were detected in PG and AG F1 offspring by PCR using the primers described above.

Ploidy analysis

Adult zebrafish were first anesthetized with tricaine (MS-222), after which approximately half of the caudal fin was excised and finely minced in 200 μL of nuclei extraction buffer (Sysmex, CyStain UV Precise P, cat# 05-5002-P02). Then the tail cells were stained with 200 μL of staining buffer containing DAPI. For embryos at 24 hpf, individual embryos were homogenized in a 1.5 mL microcentrifuge tube containing 50 μL of nuclei extraction buffer, followed by addition of 150 μL of nuclei extraction buffer. Both adult fin and embryonic samples were then stained with 200 μL of DAPI-containing staining buffer, filtered through a 40 μm cell strainer to remove debris, and

analyzed using a Beckman Cytoflex LX flow cytometer. Data acquisition and analysis were performed using CytExpert software (v2.4), with fluorescence intensity measurements used to determine DNA content and ploidy.

Gene targeting in donor cells

For gene knockout experiments, sgRNA sequences targeting *tbxta*, *smo* and *tfap2a* were adopted from published studies[23,24], while *capn3a*, *capn8* and *capn12* guide RNAs were computationally designed using CCTop (https://cctop.cos.uni-heidelberg.de/index.html) (Supplementary information, Table S6). Notably, the *capn12* guide RNAs constituted a dual-targeting system for NT-SC zebrafish generation (Supplementary information, Table S6). All sgRNAs were commercially synthesized (Azenta, China). Ribonucleoprotein (RNP) complexes were prepared on ice by combining: (1) For single-gene knock-out, 0.4 μL of 100 μM sgRNA, 0.4 μL of 10 mg/mL Cas9 protein, 0.2 μL of 10× Cas9 Buffer, 0.8 μL of DEPC-treated water and 0.2 μL of 5% phenol red (final volume 2 μL); (2) For multi-gene knockout, 0.3 μL of each 100 μM sgRNA, 0.4 μL Cas9, 0.2 μL 10× Buffer, 0.3 μL DEPC-treated water and 0.2 μL phenol red. These RNP complexes were microinjected into one-cell stage PG haploid embryos.

For gene knock-in, we designed a target site in the *huc* gene intron using CRISPR-ERA (http://crispr-era.stanford.edu/index.jsp), with sgRNA (Supplementary information, Table S6). The donor plasmid ($PCS2^{+}$) contained: (1) left homology arm spanning from upstream region of the sgRNA target site through the last exon; (2) P2A-mCherry cassette; (3) right homology arm containing the stop codon and 3′ regulatory element. The injection mixture (prepared on ice) contained: 160 ng crRNA, 160 ng tracrRNA, 30 ng donor plasmid and 0.8 μg Cas9 protein, 0.2 μL

10× Cas9 Buffer, and 0.2 μL DEPC-treated water to 2.0 μL final volume, which were microinjected into one-cell stage PG haploid embryos.

Cell cycle synchronization and analysis

For cell cycle studies, zebrafish embryos at the sphere stage (4 hpf) were collected and gently dissociated in L-15 medium (Leibovitz′s L-15). After manual removal of chorions and yolk sacs using fine forceps, the cell masses were synchronized with 2 μM nocodazole (MCE, HY-13520-10mM) in L-15 medium at 28.5°C for 1 hour. The synchronized cells were then subjected to one of the following treatments: (1) immediately utilized as nuclear transfer donors, or (2) processed for cell cycle analysis. For flow cytometric analysis, diploid and PG haploid embryos were cultured to 4 hpf at 28.5°C, followed by manual removal of chorions and yolk sacs. Cells from approximately 50 embryos were resuspended in 200 μL of nuclei extraction buffer (Sysmex, CyStain UV Precise P, cat# 05-5002-P02), then stained with 400 μL of DAPI-containing staining buffer. After filtration through a 40 μm nylon mesh, samples were analyzed using Beckman Cytoflex LX flow cytometer, with data processed through CytExpert software (v2.4).

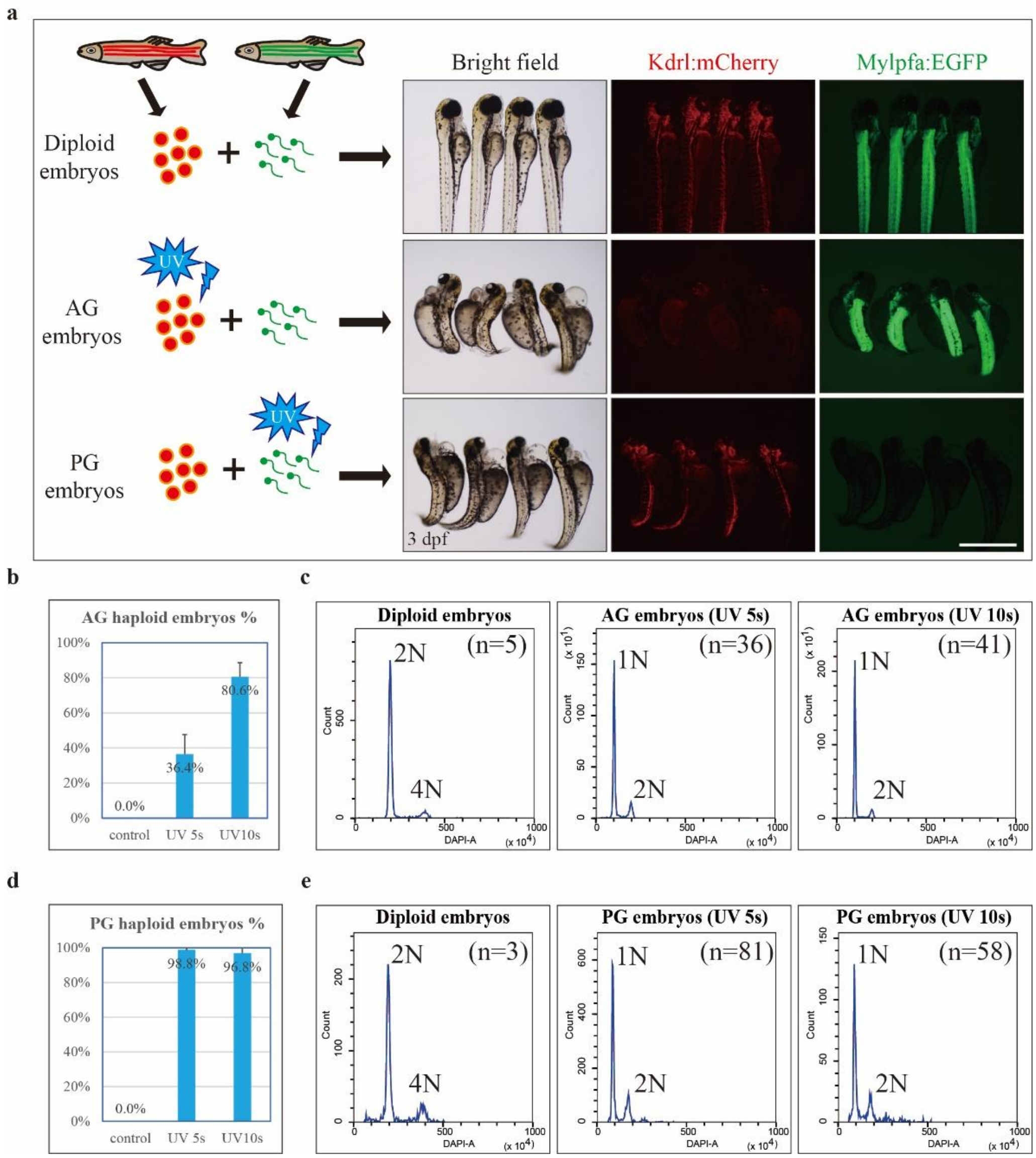

Fig. S1: Production of PG and AG haploid zebrafish embryos.

a. Generation of haploid embryos. As control, diploid embryos were produced by fertilizing eggs from Tg(*kdrl:mCherry*) females with sperm from Tg(*mylpfa:EGFP*) males, exhibiting dual fluorescence (red and green) at 3 dpf. AG haploid embryos were produced with UV-inactivated eggs and lacked red fluorescence. PG haploid embryos were generated using UV-irradiated sperm, displaying only maternal-derived red fluorescence (Kdrl:mCherry)(scale bar, 1.0 mm).

b. Efficiency of AG haploid embryo production. Comparison of AG haploid embryo yields following egg inactivation by UV irradiation for 5 and 10 s.

c. Ploidy validation of AG haploid embryos. Flow cytometry analysis of AG haploid embryos revealing distinct 1N and 2N DNA content peaks.

d. Efficiency of PG haploid embryo production. Comparison of PG haploid embryo yields following sperm inactivation by UV irradiation for 5 and 10 s.

e. Ploidy validation of PG haploid embryos. Flow cytometry analysis of PG haploid embryos revealing distinct 1N and 2N DNA content peaks.

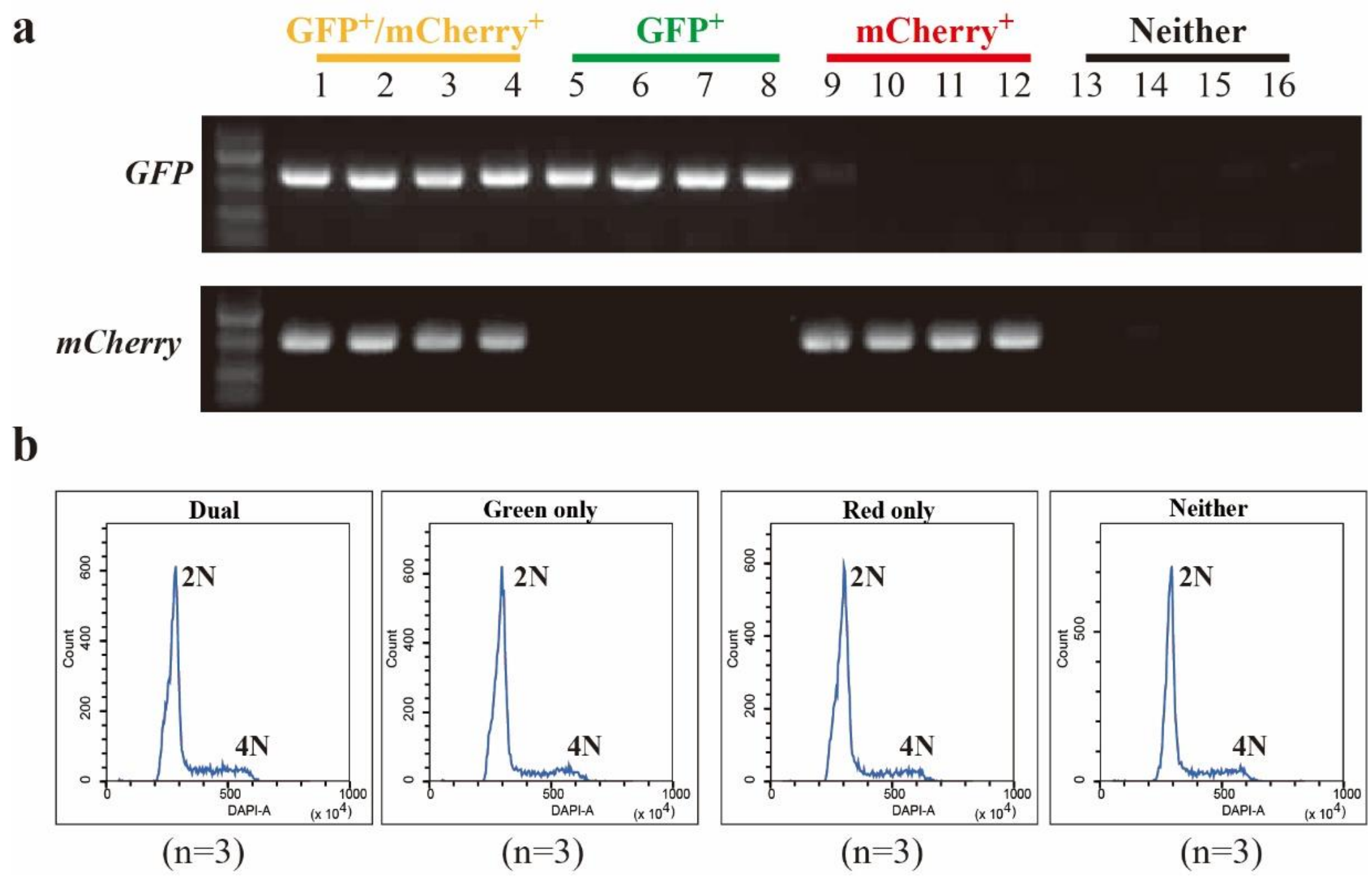

Fig. S2: Genotyping and ploidy analysis of F1 offspring from 211117SC zebrafish crossed with wild-type male.

a. Embryos exhibiting different fluorescence patterns (dual $GFP^+/mCherry^+$, GFP^+ only, $mCherry^+$ only, or no fluorescence) at 24 hpf were analyzed by PCR using specific primers for GFP and mCherry.

b. Ploidy analysis confirming diploid status across all F1 phenotypic categories, showing maintenance of euploidy through germline transmission.

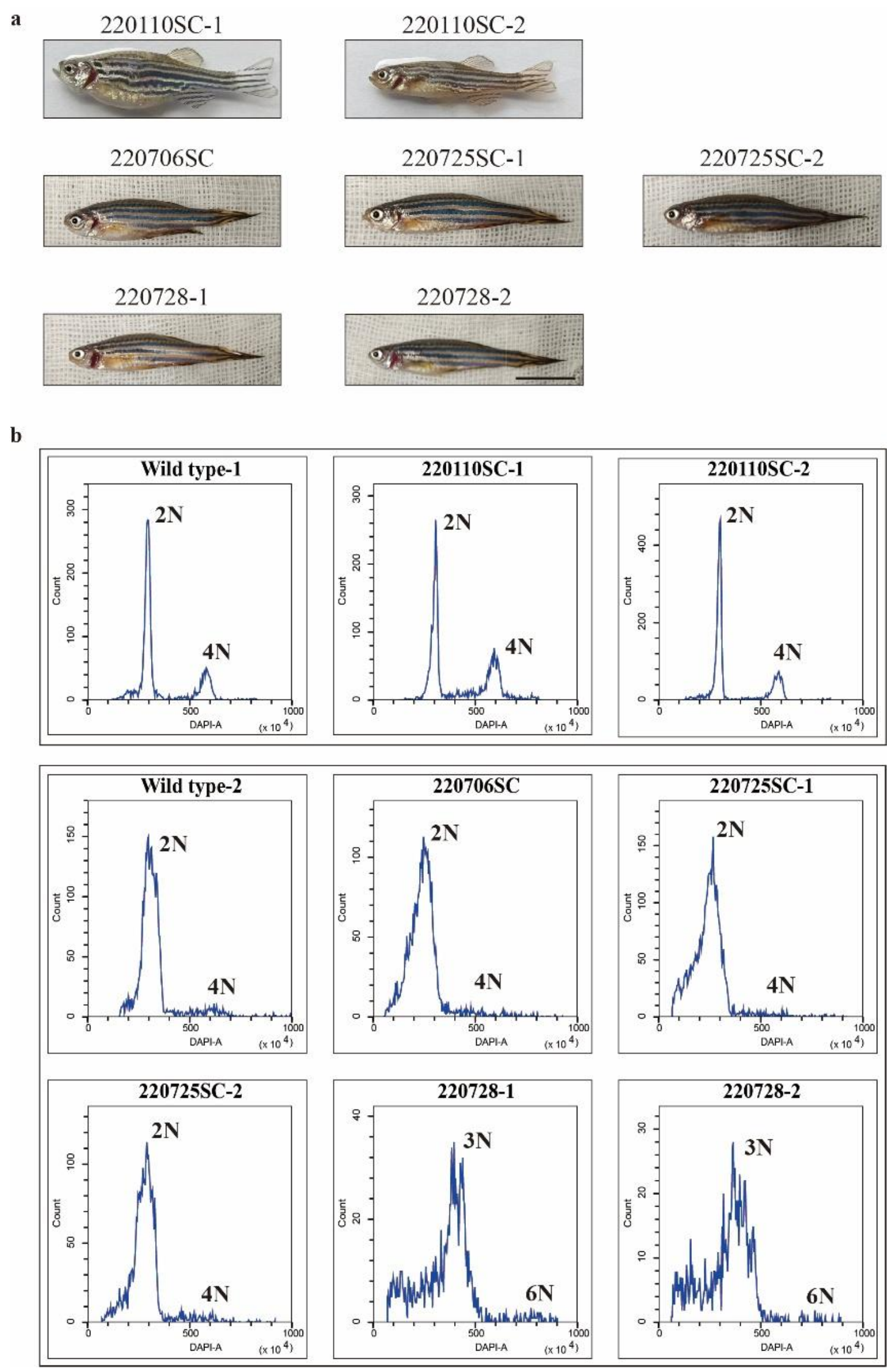

Fig. S3: Verification of adult semi-cloned zebrafish reconstructed using the piezo injection system.

a. Seven reconstructed zebrafish survived to adulthood and exhibited normal development morphology (scale bar, 1 cm).

b. FACS ploidy analysis revealed comparable DNA content profiles between wild-type and reconstructed zebrafish. Results indicated that five reconstructed fish were diploid (2N, 4N peaks) and two were triploid (3N, 6N peaks).

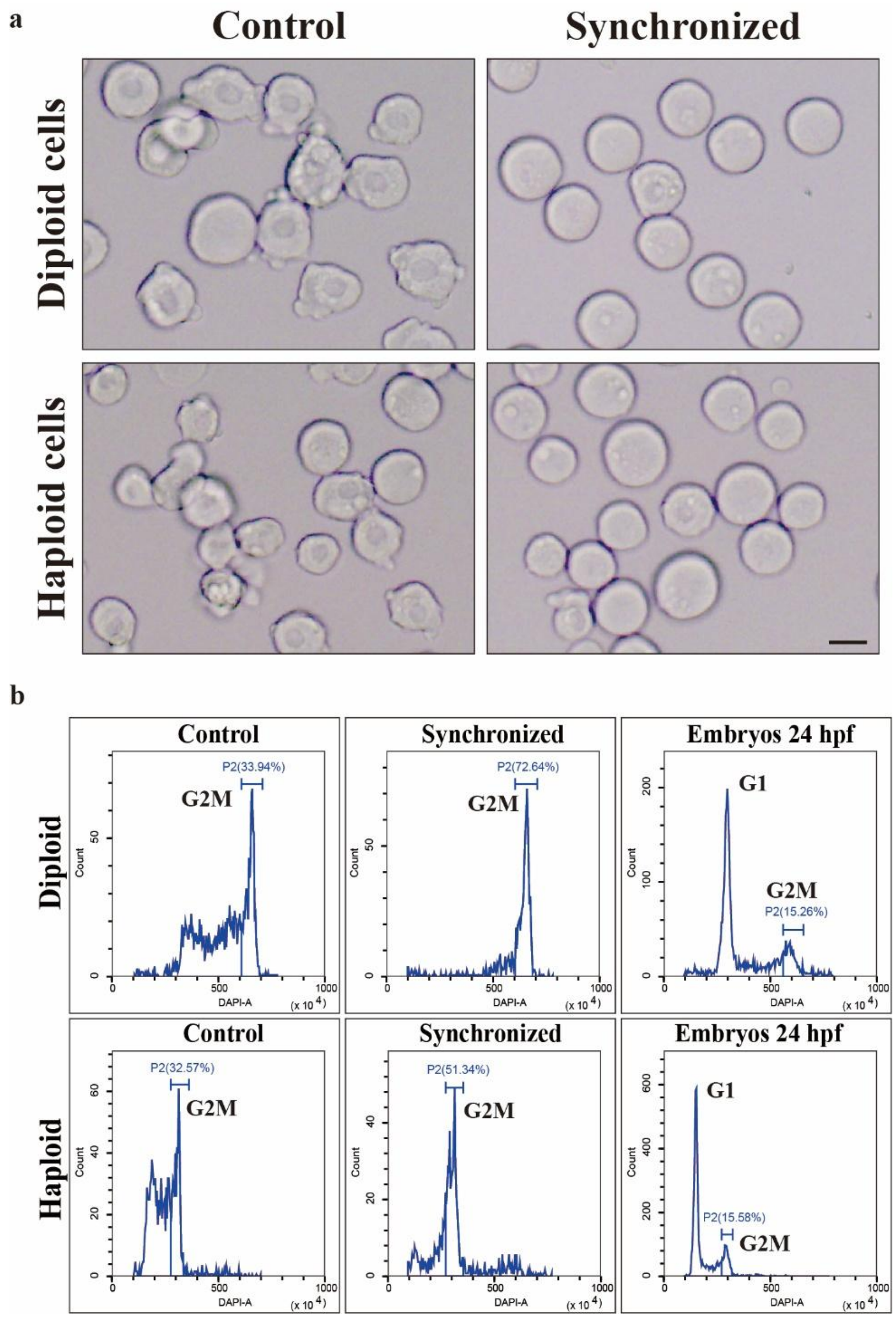

Fig. S4: The cell cycle stage analysis of nocodazole-synchronized blastula cells.

a. Morphology of synchronized blastula cells. Zebrafish blastula cells (4 hpf) were treated with 2 μM nocodazole for 1 h at 28.5°C to induce M-phase synchronization (scale bar, 20 μm).

b. Cell cycle analysis of synchronized cells. Flow cytometry (FACS) was performed to determine the cell cycle distribution following nocodazole treatment.

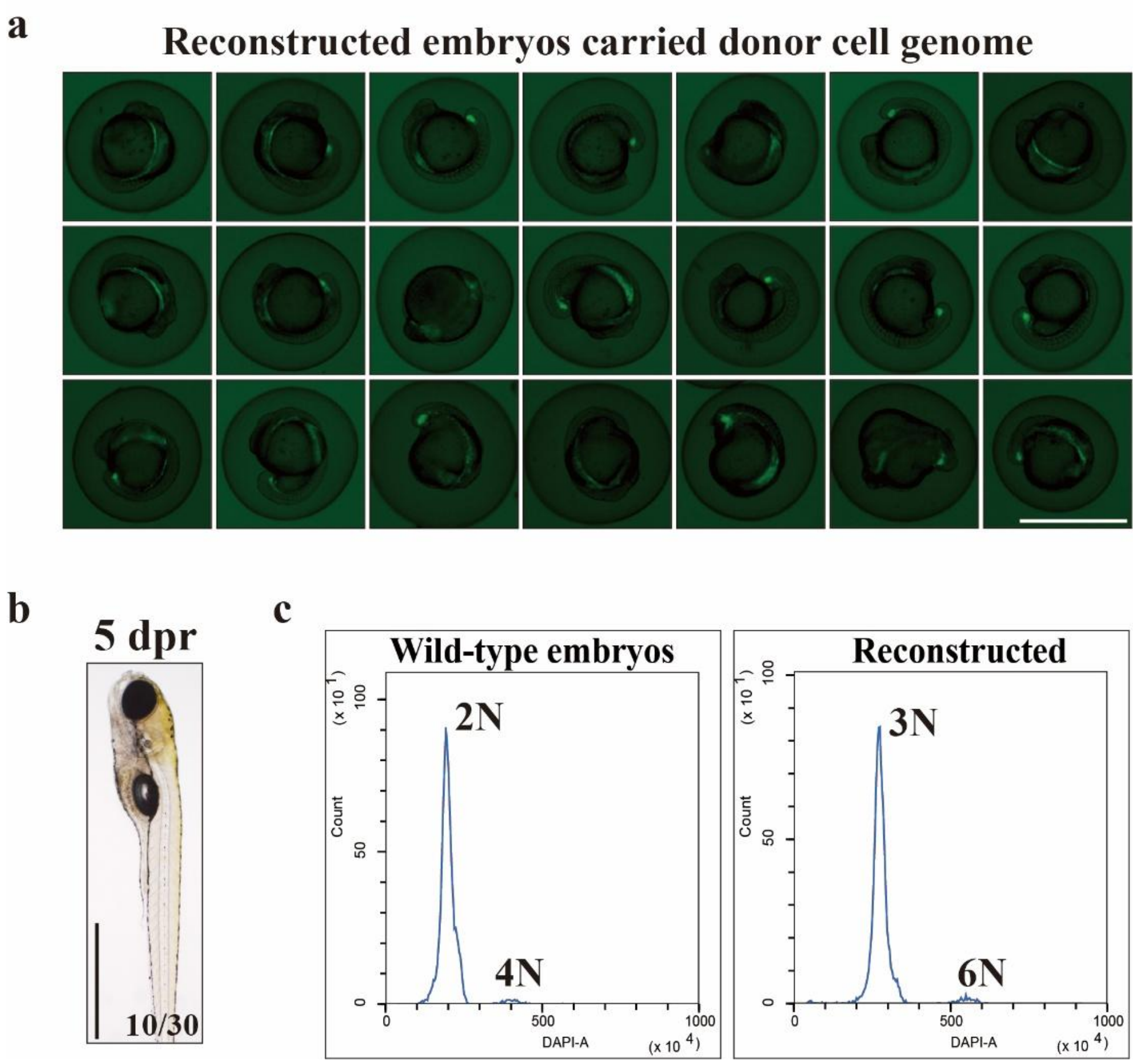

Fig. S5: Verification of reconstructed embryos derived from nocodazole-synchronized blastula cells.

a. GFP expression in reconstructed embryos. GFP expression driven by the sox17 promoter (green) confirms successful nuclear transfer in reconstructed embryos (scale bar, 1.0 mm).

b. Morphology of reconstructed embryos at 5 dpr. Reconstructed embryos displayed normal developmental morphology comparable to wild-type counterparts (scale bar, 1.0 mm).

c. Ploidy analysis of reconstructed embryos. Flow cytometry at 5 dpr revealed 9 out of 10 reconstructed embryos were triploid, with wild-type embryos serving as diploid controls.

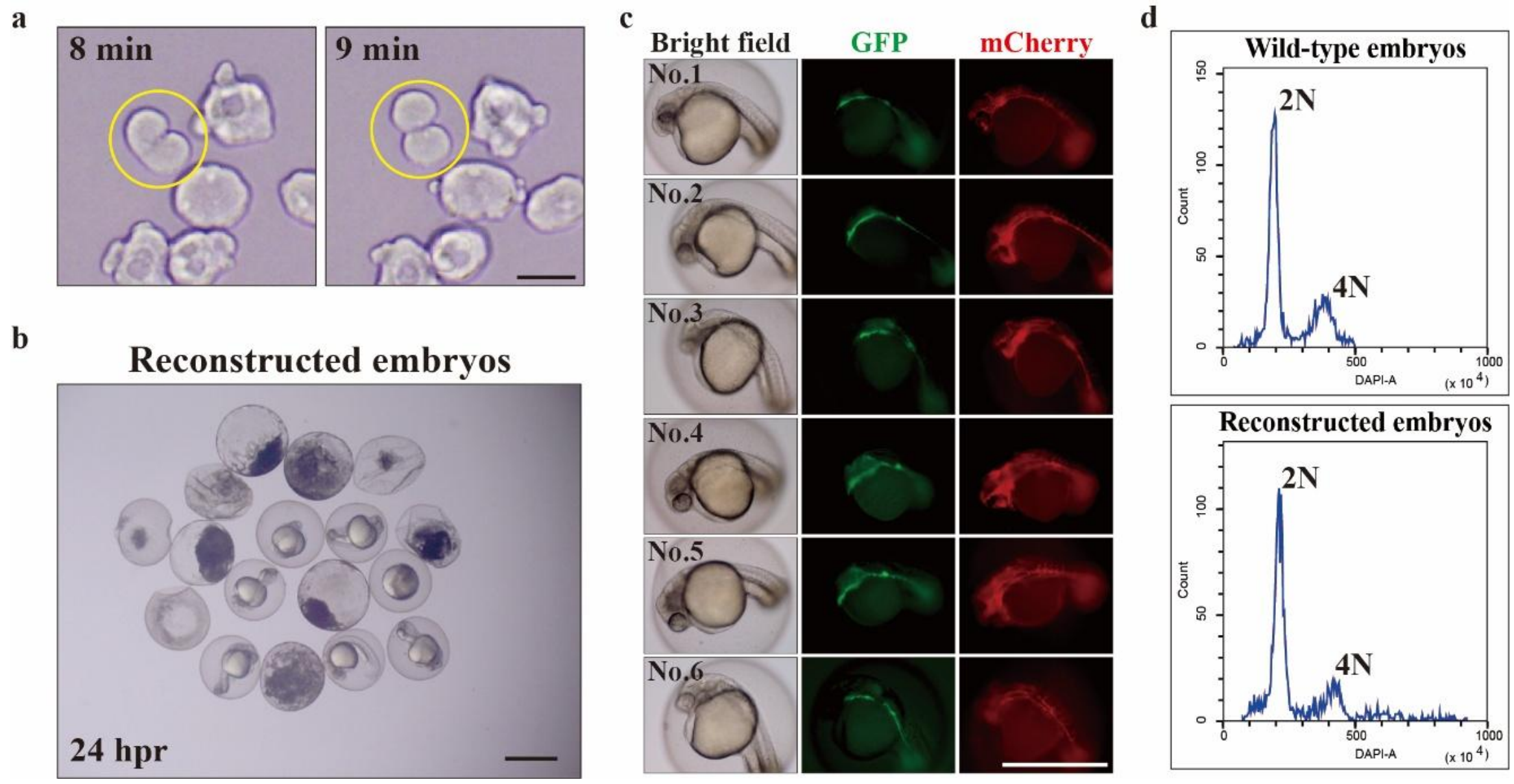

Fig. S6: Verification of reconstructed embryos derived from synchronization released telophase blastula cells.

a. Cell cycle resumption after synchronization release. Synchronized cells resumed normal mitotic division within 8 minutes after nocodazole removal (representative dividing cell indicated by yellow circle) (scale bar, 20 μm).

b. Morphology of reconstructed embryos at 24 hpr. Reconstructed embryos exhibited normal developmental progression (scale bar, 1.0 mm).

c. Fluorescence characterization of reconstructed embryos. Green fluorescence (sox17:GFP) confirmed donor cell contribution, while red fluorescence (kdrl:mCherry) marked recipient-derived tissues (scale bar, 1.0 mm).

d. Ploidy analysis of reconstructed embryos. Flow cytometry (FACS) revealed that 21 out of 26 embryos maintained diploid status.

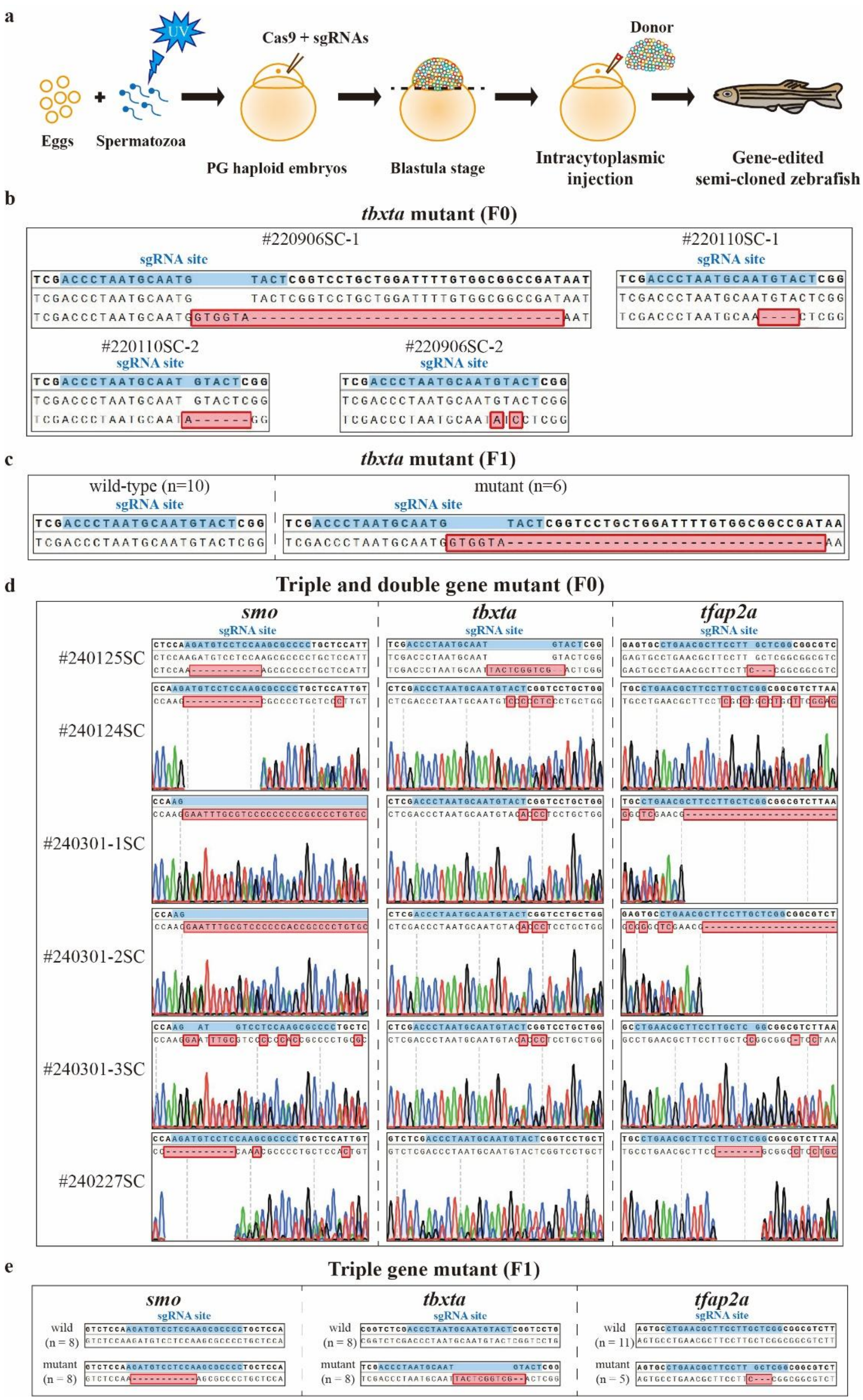

Fig. S7: Efficient generation of semi-cloned zebrafish carrying gene mutants.

a. Diagram of the one-step generation of gene-edited zebrafish. First, prepare donor embryos and inject RNP complexes (50 RNP injected embryos). When they reach the sphere stage, isolate blastula cells to generate gene-edited semi-cloned zebrafish (50 reconstructed embryos).

b. Genotyping of semi-cloned zebrafish #220906SC-1, #220110SC-1, #220110SC-2 and #220906SC-2 via TA cloning. Sanger sequencing revealed all were heterozygous, harboring the following *tbxta* mutations respectively: #220906SC-1 (27 bp indel), #220110SC-1 (4 bp deletion), #220110SC-2 (5 bp indel), #220906SC-2 (2 bp substitution) (1 male, 3 females).

c. Genotyping of F1 offspring from crossing #220906SC-1 with wild-type zebrafish. Sanger sequencing showed 10/16 samples were wild-type, while 6/16 harbored the 27 bp indel mutation.

d. Genotyping of semi-cloned zebrafish #240125SC via TA cloning. DNA sequencing revealed #240125SC was heterozygous, harboring an 11 bp deletion in *smo*, an 8 bp indel in *tbxta*, and a 2 bp indel in *tfap2a*. Semi-cloned zebrafish #240124SC, #240301-1SC, #240301-2SC, #240301-3SC and #240227SC were genotyped by Sanger sequencing of PCR products.

e. Genotyping of F1 offspring from crossing #240125SC with wild-type fish: (i) *smo*: 8/16 wild-type, 8/16 harbored the 11 bp deletion. (ii) *tbxta*: 8/16 wild-type, 8/16 harbored the 8 bp indel. (iii) *tfap2a*: 11/16 wild-type, 5/16 harbored the 2 bp indel.

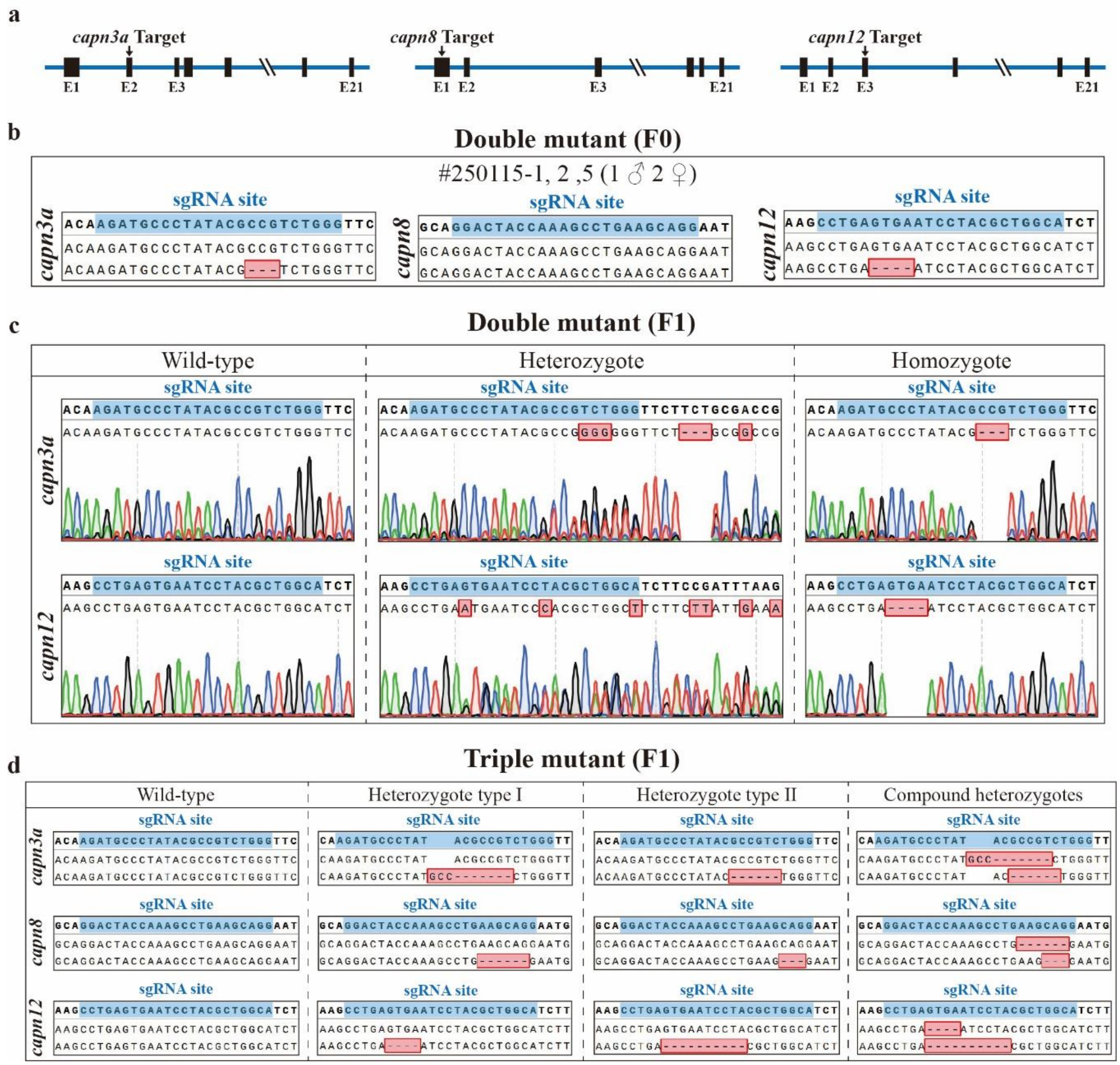

Fig. S8: Efficient generation of semi-cloned zebrafish with multiple gene mutants in a gene family.

a. Schematic of Cas9-sgRNA-targeting sites in *capn3a*, *capn8*, and *capn12*.

b. Genotyping results showing semi-cloned zebrafish #250115-1, #250115-2, and #250115-5 harbored identical mutations: a 3 bp deletion in capn3a and a 4 bp deletion in capn12.

c. Genotyping of F1 offspring from a cross between #250115-5 (male) and #250115-1/2 (female). DNA sequencing of *capn3a* in 16 embryos (1 dpf) revealed 5 wild-type, 9 heterozygous, and 2

homozygous samples. Sequencing of *capn12* revealed 5 wild-type, 8 heterozygous, and 3 homozygous samples. One sample was homozygous for both *capn3a* and *capn12*.

d. Genotyping via TA cloning of F1 offspring from a cross between #250115-4 (male) and #250113 (female). DNA sequencing of 192 embryos (1 dpf) and analysis of representative samples by TA cloning revealed: 5 wild-type, 1 triple-gene heterozygous type I, 7 triple-gene heterozygous type II, and 2 triple-gene biallelic mutation samples.

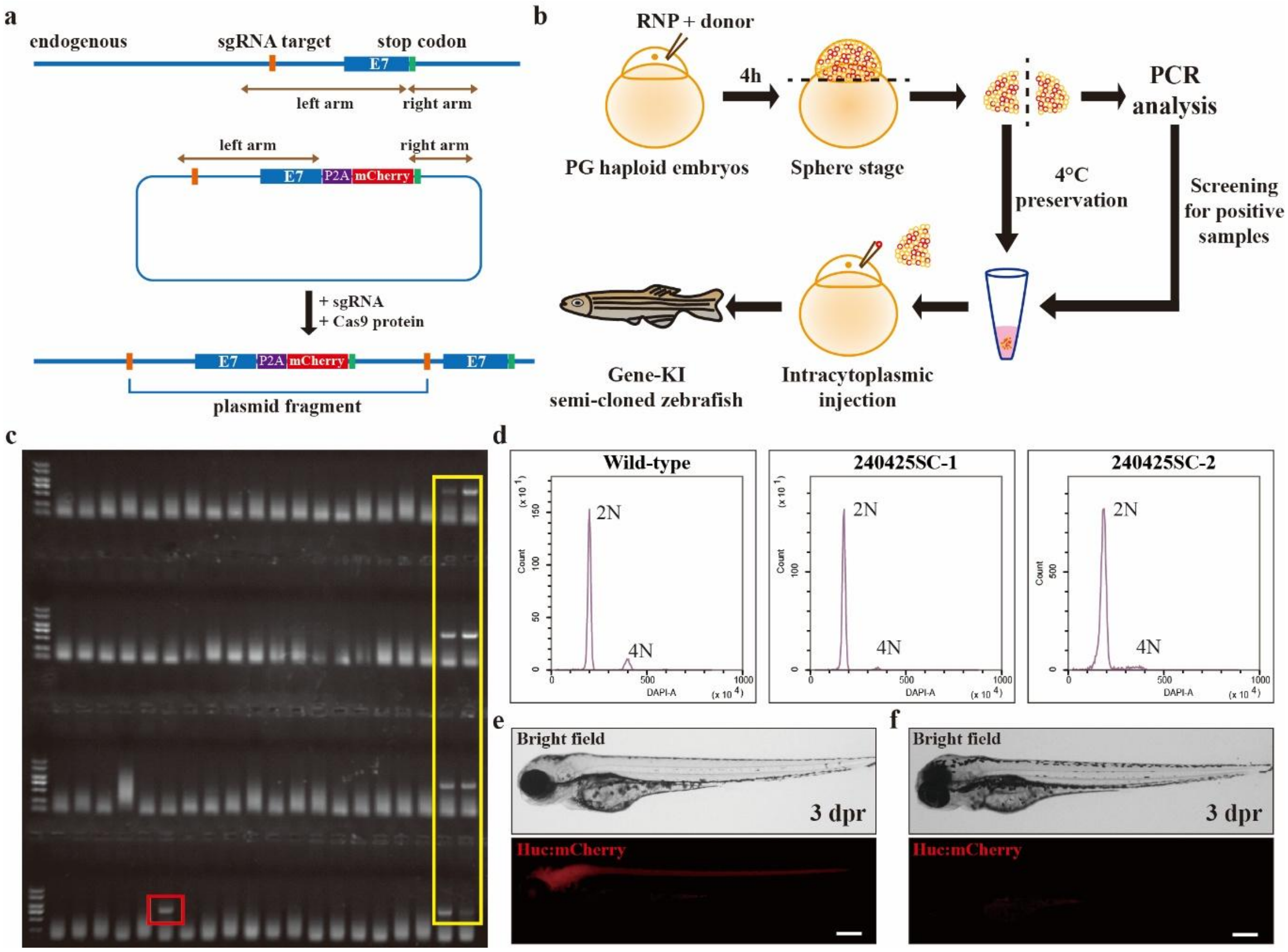

Fig. S9: Efficient generation of semi-cloned zebrafish with a gene insertion.

a. NHEJ-mediated *huc-mCherry* knock-in strategy. The target site was located in the intron upstream of the last exon of the *huc* gene. The donor plasmid contained the left homology arm, a P2A-mCherry cassette, and the right homology arm.

b. Diagram of one-step knock-in strategy. The procedure follows a similar workflow to previous methods, with donor embryos screened prior to intracytoplasmic injection (200 RNP injected embryos and 50 reconstructed embryos).

c. Agarose gel electrophoresis validation. Lanes: (i) leftmost lane: DL2000 Plus DNA marker (2000, 1500, 1000, 750, 500, 250, and 100 bp, top to bottom). (ii) yellow-boxed lanes: positive controls (785 bp) (iii) red-boxed lanes: positive knock-in sample.

d. Ploidy analysis by flow cytometry. Left: Wild-type controls showing diploid (2N) and tetraploid (4N) populations; Middle/Right: zebrafish 240425SC-1 and 240425SC-2 maintaining diploid status.

e. F1 offspring (3 dpf) exhibiting Huc:mCherry expression. Red fluorescence confirms germline transmission. Scale bar = 250 μm (scale bar, 250 μm).

f. F1 offspring (3 dpf) lacking Huc:mCherry expression (scale bar, 250 μm).

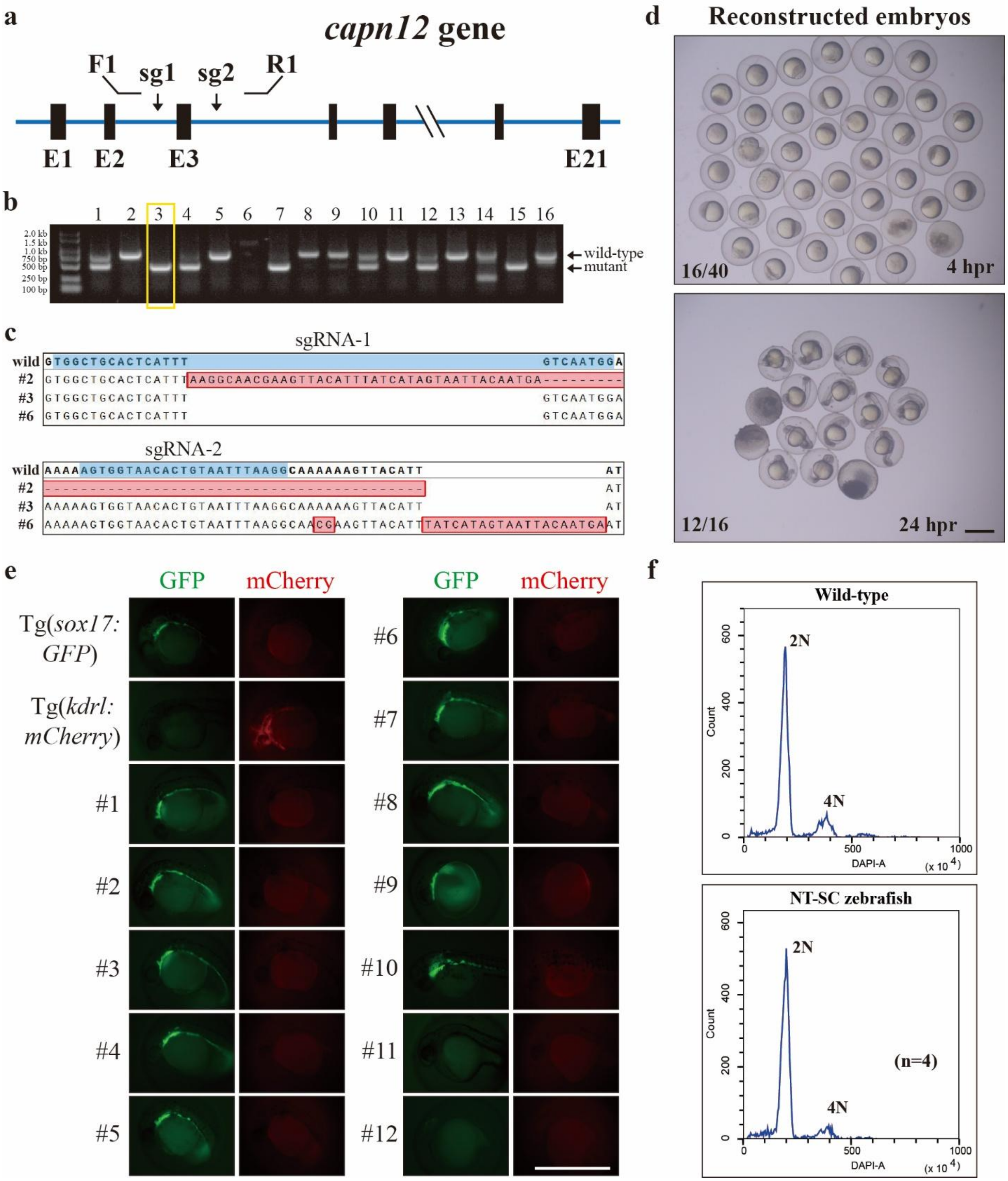

Fig. S10: Generation of semi-cloned zebrafish with a defined genotype using the NT-SC method.

a. gRNA binding sites at zebrafish *capn12* genomic locus.

b. Genotypic screening of RNP-injected PG blastula. PCR bands: WT allele (upper), mutant allele (Δ, lower). Yellow-highlighted sample selected for NT embryo preparation.

c. Sanger sequencing verification of fig.1k amplicons, verifying precise deletion in mutant alleles. #2 sample proceeded to NT-SC embryo generation.

d. Nuclear reprogramming: Representative 4 hpr and 24 hpr embryos exhibited typical morphology (scale bar, 1.0 mm).

e. Fluorescence validation: GFP expression confirmed donor-derived nuclear content in reconstructed embryos. Absence of mCherry expression excluded residual maternal DNA from UV-inactivated host eggs (scale bar, 1.0 mm).

f. Adult NT-SC zebrafish ploidy validation. FACS ploidy analysis showing comparable DNA content profiles between wild-type fish and NT-SC zebrafish, both exhibiting diploid (2N) and tetraploid (4N) peaks.

Table S1. Development of semi-cloned embryos reconstructed from freshly isolated haploid blastula cells using two different operation systems

System	Donor Cell Line	Recipient egg	No. of reconstructed Embryos	Blastula (% of reconstructed Embryos)	Epiboly (% of reconstructed Embryos)	1 dpr (% of reconstructed Embryos)		3 dpr (% of reconstructed Embryos)	5 dpr (% of reconstructed Embryos)	Adult (% of reconstructed Embryos)	
						Carried donor genome	Parthenogenesis			Diploid	Triploid
pneumatic operation system	Tg(*mylpfa:EGFP*)-AG	AB	20	11 (55.0)	4 (20.0)	1 (5.0)	2 (10.0)	1 (5.0)	0	0	0
	Tg(*sox17:GFP*)-AG	AB	52	42 (80.8)	13 (25.0)	4 (7.7)	2 (3.8)	3 (5.8)	3 (5.8)	1 (2.0)	0
		Tg(*kdrl:mCherry*)	31	15 (48.4)	4 (12.9)	1 (3.2)	0	1 (0.9)	0	0	0
	Tg(*fli1:EGFP*)-AG	AB	280	149 (53.2)	40 (14.3)	5 (1.8)	5 (1.8)	4 (1.4)	2 (0.7)	0	0
		Tg(*kdrl:mCherry*)	322	209 (64.9)	58 (18.0)	8 (2.5)	12 (3.7)	6 (1.9)	6 (1.9)	1 (0.3)	1 (0.3)
	Tg(*kdrl:mCherry*)-PG	Tg(*mylpfa:EGFP*)	226	105 (46.5)	53 (23.5)	5 (2.2)	20 (8.8)	5 (2.2)	5 (2.2)	0	0
subtotal			931	531 (57.0)	172 (18.5)	24 (2.6)	41 (4.4)	20 (2.1)	16 (1.7)	2 (0.2)	1 (0.1)
piezo system	Tg(*kdrl:mCherry*)-PG	AB	21	19 (90.5)	9 (42.9)	1 (4.8)	2 (9.5)	1 (4.8)	1 (4.8)	0	0
		Tg(*sox17:GFP*)	68	48 (70.6)	33 (48.5)	7 (10.3)	9 (13.2)	7 (10.3)	5 (7.4)	1 (1.5)	0
	Tg(*sox17:GFP*)-PG	Tg(*kdrl:mCherry*)	56	40 (71.4)	31 (55.4)	11 (19.6)	4 (7.1)	11 (19.6)	11 (19.6)	2 (3.6)	2 (3.6)
	Tg(*fli1:EGFP*)-PG	Tg(*kdrl:mCherry*)	22	17 (77.3)	9 (40.9)	4 (18.2)	1 (4.5)	4 (18.2)	3 (13.6)	2 (9.1)	0
subtotal			167	124 (74.3)	82 (49.1)	23 (13.8)	16 (9.6)	23 (13.8)	20 (12.0)	5 (3.0)	2 (1.2)

Table S2. Development of semi-cloned embryos derived from freshly isolated haploid blastula cells with different cell cycle stages

Temperature program	Cell cycle stage of donor cells	No. of reconstructed embryos	1 dpr (% of reconstructed embryos)		5 dpr (% of reconstructed embryos)		Adult (% of reconstructed embryos)	
			Carried parental genomes	PG & AG	Normal	Abnormal	Diploid	Triploid
Step1. operation at 24°C<br>Step2. 1h-culture at 20°C<br>Step3. culture to adult at 28°C	G1/S	103	16 (15.5)	24 (23.3)	3 (2.9)	9 (8.7)	2 (1.9)	1 (1.0)
	G2/M	101	15 (14.9)	3 (3.0)	3 (3.0)	7 (6.9)	1 (1.0)	3 (3.0)
	Telophase	108	56 (51.9)	8 (7.4)	38 (35.2)	12 (11.1)	33 (30.6)	1 (1.6)
Step1. operation at 20°C<br>Step2. 1h-culture at 20°C<br>Step3. culture to adult at 28°C	G1/S	98	12 (12.2)	21 (21.1)	2 (2.0)	6 (6.1)	1 (1.0)	1 (1.0)
	G2/M	107	19 (17.8)	2 (1.9)	7 (6.5)	7 (6.5)	0	5 (4.7)
	Telophase	136	60 (44.1)	12 (8.8)	32 (23.5)	21 (15.4)	22 (16.2)	3 (2.2)
Step1. operation at 24°C<br>Step2. 1h-culture at 24°C<br>Step3. culture to adult at 28°C	G1/S	108	25 (23.1)	17 (15.7)	8 (7.4)	6 (5.6)	6 (5.6)	1 (0.9)
	G2/M	101	9 (8.9)	1 (10.)	4 (4.0)	3 (3.0)	0	4 (4.0)
	Telophase	111	49 (44.1)	16 (14.4)	36 (32.4)	5 (4.5)	25 (22.5)	4 (3.6)
Step1. operation at 24°C<br>Step2. culture to adult at 28°C	G1/S	94	9 (9.6)	12 (12.8)	1 (1.1)	5 (2.3)	1 (1.1)	0
	G2/M	102	6 (5.9)	0	1 (1.0)	2 (2.0)	0	1 (9.8)
	Telophase	103	16 (15.5)	8 (7.8)	8 (7.8)	6 (5.8)	7 (6.8)	1 (1.0)

Table S3. Summary of semi-cloned zebrafish generated in this study

No.	Fish code	Sex	Ploidy	Fertility	Donor	Receptor	Genotype
1	211013SC	female	diploid	yes	AG (Tg(*sox17:GFP*))	AB	
2	211117SC	female	diploid	yes	AG (Tg(*fli1:EGFP*))	Tg(*kdrl:mCherry*)	
3	211125	male	ND	no	AG (Tg(fli1:EGFP))	kdrl-mCherry	
4	220110SC-1	female	diploid	yes	PG (Tg(*fli1:EGFP*))	Tg(*kdrl:mCherry*)	*tbxta* $^{+/-}$
5	220110SC-2	male	diploid	yes	PG (Tg(*fli1:EGFP*))	Tg(*kdrl:mCherry*)	*tbxta* $^{+/-}$
6	220706SC-1	male	diploid	yes	PG (Tg(*kdrl:mCherry*))	Tg(*sox17:GFP*)	
7	220725SC-1	male	diploid	yes	PG (Tg(*sox17:GFP*))	Tg(*kdrl:mCherry*)	
8	220725SC-2	male	diploid	yes	PG (Tg(*sox17:GFP*))	Tg(*kdrl:mCherry*)	
9	220728-1	male	triploid	ND	PG (sox17-GFP)	kdrl-mCherry	
10	220728-2	male	triploid	low	PG (sox17-GFP)	kdrl-mCherry	
11	220829SC	male	diploid	yes	PG (Tg(*kdrl:mCherry*))	Tg(*sox17:GFP*)	
12	220906SC-1	female	diploid	yes	PG (Tg(*kdrl:mCherry*))	Tg(*sox17:GFP*)	*tbxta* $^{+/-}$
13	220906SC-2	female	diploid	yes	PG (Tg(*kdrl:mCherry*))	Tg(*sox17:GFP*)	*tbxta* $^{+/-}$
14	230511	male	diploid	yes	PG (Tg(*sox17:GFP*))	Tg(*kdrl:mCherry*)	
15	230601	male	diploid	yes	PG (Tg(*sox17:GFP*))	AB	
16	230731-1	female	diploid	yes	PG (Tg(*sox17:GFP*))	AB	
17	230731-2	female	diploid	yes	PG (Tg(*sox17:GFP*))	AB	
18	230731-3	female	diploid	yes	PG (Tg(*sox17:GFP*))	AB	
19	230731-4	female	diploid	yes	PG (Tg(*sox17:GFP*))	AB	
20	230731-5	female	diploid	yes	PG (Tg(*sox17:GFP*))	AB	
21	230731-8	female	diploid	yes	PG (Tg(*sox17:GFP*))	AB	
22	230731-9	female	diploid	yes	PG (Tg(*sox17:GFP*))	AB	
23	230816	male	diploid	yes	PG (Tg(*sox17:GFP*))	AB	
24	230829-2	female	diploid	yes	PG (Tg(*sox17:GFP*))	AB	
25	230831	male	diploid	yes	PG (Tg(*sox17:GFP*))	AB	
26	240124	female	diploid	ND	PG (Tg(*sox17:GFP*))	AB	*smo* $^{+/-}$, *tbxta* $^{+/-}$, *tfap2a* $^{+/-}$
27	240125	male	diploid	yes	PG (Tg(*sox17:GFP*))	AB	*smo* $^{+/-}$, *tbxta* $^{+/-}$, *tfap2a* $^{+/-}$
28	240227	male	diploid	yes	PG (Tg(*sox17:GFP*))	AB	*smo* $^{+/-}$, *tfap2a* $^{+/-}$
29	240301-1	female	diploid	yes	PG (Tg(*sox17:GFP*))	AB	*smo* $^{+/-}$, *tbxta* $^{+/-}$, *tfap2a* $^{+/-}$
30	240301-2	female	diploid	yes	PG (Tg(*sox17:GFP*))	AB	*smo* $^{+/-}$, *tbxta* $^{+/-}$, *tfap2a* $^{+/-}$
31	240301-3	female	diploid	yes	PG (Tg(*sox17:GFP*))	AB	*smo* $^{+/-}$, *tbxta* $^{+/-}$, *tfap2a* $^{+/-}$
32	24031415-1	male	diploid	yes	PG (Tg(*sox17:GFP*))	AB	
33	24031415-2	male	diploid	yes	PG (Tg(*sox17:GFP*))	AB	
34	24032021-1	female	diploid	yes	PG (Tg(*sox17:GFP*))	AB	
35	24032021-2	female	diploid	yes	PG (Tg(*sox17:GFP*))	AB	
36	24032021-3	female	diploid	yes	PG (Tg(*sox17:GFP*))	AB	
37	24032021-4	male	diploid	yes	PG (Tg(*sox17:GFP*))	AB	
38	24032021-5	male	diploid	yes	PG (Tg(*sox17:GFP*))	AB	
39	240403-2	male	diploid	yes	PG (Tg(*sox17:GFP*))	AB	
40	240405-1	female	diploid	yes	PG (Tg(*sox17:GFP*))	AB	
41	240405-2	female	diploid	yes	PG (Tg(*sox17:GFP*))	AB	
42	240405-3	female	diploid	yes	PG (Tg(*sox17:GFP*))	AB	
43	240405-4	male	diploid	ND	PG (Tg(*sox17:GFP*))	AB	
44	240410	female	diploid	yes	PG (Tg(*sox17:GFP*))	AB	
45	24041719-1	female	diploid	yes	PG (Tg(*sox17:GFP*))	AB	
46	24041719-2	female	diploid	yes	PG (Tg(*sox17:GFP*))	AB	
47	24041719-3	female	diploid	yes	PG (Tg(*sox17:GFP*))	AB	
48	24041719-4	female	diploid	yes	PG (Tg(*sox17:GFP*))	AB	

49	24041719-5	female	diploid	yes	PG (Tg(*sox17:GFP*))	AB	
50	240425-1	female	diploid	yes	PG (Tg(*sox17:GFP*))	AB	*huc-mCherry* $^{+/-}$
51	240425-2	female	diploid	yes	PG (Tg(*sox17:GFP*))	AB	*huc-mCherry* $^{+/-}$
52	240425-3	female	diploid	yes	PG (Tg(*sox17:GFP*))	AB	
53	240425-4	female	diploid	yes	PG (Tg(*sox17:GFP*))	AB	
54	24112021-1	female	diploid	yes	PG (Tg(*kdrl:mCherry*))	Tg(*sox17:GFP*)	*capn3a* $^{+/-}$, *capn12* $^{+/-}$
55	24112021-2	female	diploid	yes	PG (Tg(*kdrl:mCherry*))	Tg(*sox17:GFP*)	*capn3a* $^{+/-}$, *capn12* $^{+/-}$
56	250107	female	diploid	ND	PG (Tg(*kdrl:mCherry*))	AB	
57	250113	female	diploid	yes	PG (Tg(*kdrl:mCherry*))	Tg(*sox17:GFP*)	*capn3a* $^{+/-}$, *capn8* $^{+/-}$, *capn12* $^{+/-}$
58	250115-1	female	diploid	yes	PG (Tg(*kdrl:mCherry*))	Tg(*sox17:GFP*)	*capn3a* $^{+/-}$, *capn12* $^{+/-}$
59	250115-2	female	diploid	yes	PG (Tg(*kdrl:mCherry*))	Tg(*sox17:GFP*)	*capn3a* $^{+/-}$, *capn12* $^{+/-}$
60	250115-3	male	diploid	yes	PG (Tg(*kdrl:mCherry*))	Tg(*sox17:GFP*)	*capn3a* $^{+/-}$, *capn12* $^{+/-}$
61	250115-4	male	diploid	yes	PG (Tg(*kdrl:mCherry*))	Tg(*sox17:GFP*)	*capn3a* $^{+/-}$, *capn8* $^{+/-}$, *capn12* $^{+/-}$
62	250115-5	male	diploid	yes	PG (Tg(*kdrl:mCherry*))	Tg(*sox17:GFP*)	*capn3a* $^{+/-}$, *capn12* $^{+/-}$
63	250115-6	male	diploid	yes	PG (Tg(*kdrl:mCherry*))	Tg(*sox17:GFP*)	*capn3a* $^{+/-}$, *capn12* $^{-/-}$
64	250214-G	male	diploid	yes	PG (Tg(*sox17:GFP*))	Tg(*kdrl:mCherry*)	
65	250214-T-1	male	diploid	yes	PG (Tg(*sox17:GFP*))	Tg(*kdrl:mCherry*)	
66	250214-T-2	female	diploid	yes	PG (Tg(*sox17:GFP*))	Tg(*kdrl:mCherry*)	
67	250214-T-3	female	diploid	yes	PG (Tg(*sox17:GFP*))	Tg(*kdrl:mCherry*)	
68	250214-T-4	female	diploid	yes	PG (Tg(*sox17:GFP*))	Tg(*kdrl:mCherry*)	
69	250214-T-5	female	diploid	yes	PG (Tg(*sox17:GFP*))	Tg(*kdrl:mCherry*)	
70	250214-T-6	female	diploid	ND	PG (Tg(*sox17:GFP*))	Tg(*kdrl:mCherry*)	
71	250217-T-1	female	diploid	yes	PG (Tg(*sox17:GFP*))	Tg(*kdrl:mCherry*)	
72	250217-T-2	female	diploid	yes	PG (Tg(*sox17:GFP*))	Tg(*kdrl:mCherry*)	
73	250218-T-1	female	diploid	yes	PG (Tg(*sox17:GFP*))	Tg(*kdrl:mCherry*)	
74	250218-T-2	female	diploid	yes	PG (Tg(*sox17:GFP*))	Tg(*kdrl:mCherry*)	
75	250218-T-3	female	diploid	yes	PG (Tg(*sox17:GFP*))	Tg(*kdrl:mCherry*)	
76	250218-T-4	male	diploid	yes	PG (Tg(*sox17:GFP*))	Tg(*kdrl:mCherry*)	
77	250218-T-5	male	diploid	yes	PG (Tg(*sox17:GFP*))	Tg(*kdrl:mCherry*)	
78	250218-T-6	female	diploid	yes	PG (Tg(*sox17:GFP*))	Tg(*kdrl:mCherry*)	
79	250218-T-7	male	diploid	yes	PG (Tg(*sox17:GFP*))	Tg(*kdrl:mCherry*)	
80	250218-T-8	female	diploid	yes	PG (Tg(*sox17:GFP*))	Tg(*kdrl:mCherry*)	
81	250218-T-9	male	diploid	yes	PG (Tg(*sox17:GFP*))	Tg(*kdrl:mCherry*)	
82	250218-T-10	male	diploid	ND	PG (Tg(*sox17:GFP*))	Tg(*kdrl:mCherry*)	
83	250219-G	female	diploid	yes	PG (Tg(*sox17:GFP*))	Tg(*kdrl:mCherry*)	
84	250219-M	female	diploid	yes	PG (Tg(*sox17:GFP*))	Tg(*kdrl:mCherry*)	
85	250220-T	female	diploid	yes	PG (Tg(*sox17:GFP*))	Tg(*kdrl:mCherry*)	
86	250221-T-1	female	diploid	yes	PG (Tg(*sox17:GFP*))	Tg(*kdrl:mCherry*)	
87	250221-T-1	female	diploid	yes	PG (Tg(*sox17:GFP*))	Tg(*kdrl:mCherry*)	
88	250224-T	male	diploid	yes	PG (Tg(*sox17:GFP*))	Tg(*kdrl:mCherry*)	
89	250225-T-1	male	diploid	yes	PG (Tg(*sox17:GFP*))	Tg(*kdrl:mCherry*)	
90	250225-T-2	female	diploid	yes	PG (Tg(*sox17:GFP*))	Tg(*kdrl:mCherry*)	
91	250225-T-3	female	diploid	yes	PG (Tg(*sox17:GFP*))	Tg(*kdrl:mCherry*)	
92	250225-T-4	male	diploid	yes	PG (Tg(*sox17:GFP*))	Tg(*kdrl:mCherry*)	
93	250226-T	female	diploid	yes	PG (Tg(*sox17:GFP*))	Tg(*kdrl:mCherry*)	
94	250227-T-1	female	diploid	yes	PG (Tg(*sox17:GFP*))	Tg(*kdrl:mCherry*)	
95	250227-T-2	female	diploid	ND	PG (Tg(*sox17:GFP*))	Tg(*kdrl:mCherry*)	
96	250304-T-1	female	diploid	yes	PG (Tg(*sox17:GFP*))	Tg(*kdrl:mCherry*)	
97	250304-T-2	female	diploid	yes	PG (Tg(*sox17:GFP*))	Tg(*kdrl:mCherry*)	
98	250307-T	male	diploid	ND	PG (Tg(*sox17:GFP*))	Tg(*kdrl:mCherry*)	

99	250317-T-1	female	diploid	yes	PG (Tg(*sox17:GFP*))	Tg(*kdrl:mCherry*)	
100	250317-T-2	female	diploid	yes	PG (Tg(*sox17:GFP*))	Tg(*kdrl:mCherry*)	
101	250331-T-1	female	diploid	yes	PG (Tg(*sox17:GFP*))	Tg(*kdrl:mCherry*)	
102	250331-T-2	male	ND	yes	PG (Tg(*sox17:GFP*))	Tg(*kdrl:mCherry*)	
103	250401-T-1	female	diploid	yes	PG (Tg(*sox17:GFP*))	Tg(*kdrl:mCherry*)	
104	250401-T-2	female	diploid	yes	PG (Tg(*sox17:GFP*))	Tg(*kdrl:mCherry*)	
105	250401-T-3	female	diploid	yes	PG (Tg(*sox17:GFP*))	Tg(*kdrl:mCherry*)	
106	250401-T-4	female	diploid	yes	PG (Tg(*sox17:GFP*))	Tg(*kdrl:mCherry*)	
107	250407-T-1	female	diploid	yes	PG (Tg(*sox17:GFP*))	Tg(*kdrl:mCherry*)	
108	250407-T-2	female	diploid	yes	PG (Tg(*sox17:GFP*))	Tg(*kdrl:mCherry*)	
109	250408-T-1	female	diploid	yes	PG (Tg(*sox17:GFP*))	Tg(*kdrl:mCherry*)	
110	250408-T-2	female	diploid	yes	PG (Tg(*sox17:GFP*))	Tg(*kdrl:mCherry*)	
111	250408-T-3	female	diploid	yes	PG (Tg(*sox17:GFP*))	Tg(*kdrl:mCherry*)	
112	250408-T-4	female	diploid	yes	PG (Tg(*sox17:GFP*))	Tg(*kdrl:mCherry*)	
113	250408-T-5	female	diploid	yes	PG (Tg(*sox17:GFP*))	Tg(*kdrl:mCherry*)	
114	250408-T-6	female	diploid	yes	PG (Tg(*sox17:GFP*))	Tg(*kdrl:mCherry*)	
115	250409-T	female	diploid	yes	PG (Tg(*sox17:GFP*))	Tg(*kdrl:mCherry*)	
116	250415-T	male	diploid	yes	PG (Tg(*sox17:GFP*))	Tg(*kdrl:mCherry*)	
117	250417-T	male	diploid	yes	PG (Tg(*sox17:GFP*))	Tg(*kdrl:mCherry*)	
118	250424-G	female	diploid	yes	PG (Tg(*sox17:GFP*))	Tg(*kdrl:mCherry*)	
119	250425-G	female	diploid	yes	PG (Tg(*sox17:GFP*))	Tg(*kdrl:mCherry*)	
120	250426-1	female	diploid	yes	NT (Tg(*sox17:GFP*))	AB	*capn12* $^{+/-}$
121	250426-2	female	diploid	yes	NT (Tg(*sox17:GFP*))	AB	*capn12* $^{+/-}$
122	250426-3	male	diploid	yes	NT (Tg(*sox17:GFP*))	AB	*capn12* $^{+/-}$
123	250426-4	male	diploid	yes	NT (Tg(*sox17:GFP*))	AB	*capn12* $^{+/-}$

124	1-T-0106-1	female	diploid	yes	PG (Tg(*sox17:GFP*))	Tg(*kdrl:mCherry*)
125	1-T-0106-2	female	diploid	ND	PG (Tg(*sox17:GFP*))	Tg(*kdrl:mCherry*)
126	1-T-0108-1	female	diploid	yes	PG (Tg(*sox17:GFP*))	Tg(*kdrl:mCherry*)
127	1-T-0108-2	female	diploid	yes	PG (Tg(*sox17:GFP*))	Tg(*kdrl:mCherry*)
128	1-T-0108-3	female	diploid	yes	PG (Tg(*sox17:GFP*))	Tg(*kdrl:mCherry*)
129	1-T-0108-4	female	diploid	yes	PG (Tg(*sox17:GFP*))	Tg(*kdrl:mCherry*)
130	1-T-0112-1	female	diploid	yes	PG (Tg(*sox17:GFP*))	Tg(*kdrl:mCherry*)
131	1-T-0112-2	female	diploid	yes	PG (Tg(*sox17:GFP*))	Tg(*kdrl:mCherry*)
132	1-T-0112-3	female	diploid	yes	PG (Tg(*sox17:GFP*))	Tg(*kdrl:mCherry*)
133	1-T-0112-4	male	diploid	yes	PG (Tg(*sox17:GFP*))	Tg(*kdrl:mCherry*)
134	1-T-0119-1	female	diploid	yes	PG (Tg(*sox17:GFP*))	Tg(*kdrl:mCherry*)
135	1-T-0119-2	female	diploid	yes	PG (Tg(*sox17:GFP*))	Tg(*kdrl:mCherry*)
136	2-T-0122-1	female	diploid	yes	PG (Tg(*sox17:GFP*))	Tg(*kdrl:mCherry*)
137	2-T-0122-2	female	diploid	yes	PG (Tg(*sox17:GFP*))	Tg(*kdrl:mCherry*)
138	2-T-0122-3	female	diploid	yes	PG (Tg(*sox17:GFP*))	Tg(*kdrl:mCherry*)
139	2-T-0202-1	female	diploid	yes	PG (Tg(*sox17:GFP*))	Tg(*kdrl:mCherry*)
140	2-T-0202-2	female	diploid	yes	PG (Tg(*sox17:GFP*))	Tg(*kdrl:mCherry*)
141	2-T-0203-1	female	diploid	yes	PG (Tg(*sox17:GFP*))	Tg(*kdrl:mCherry*)
142	2-T-0203-2	female	diploid	yes	PG (Tg(*sox17:GFP*))	Tg(*kdrl:mCherry*)
143	2-T-0203-3	female	diploid	yes	PG (Tg(*sox17:GFP*))	Tg(*kdrl:mCherry*)
144	2-G1S-0203-1	male	diploid	ND	PG (Tg(*sox17:GFP*))	Tg(*kdrl:mCherry*)
145	2-G1S-0203-2	female	diploid	yes	PG (Tg(*sox17:GFP*))	Tg(*kdrl:mCherry*)
146	2-G1S-0203-3	male	diploid	yes	PG (Tg(*sox17:GFP*))	Tg(*kdrl:mCherry*)
147	2-G1S-0203-4	male	diploid	yes	PG (Tg(*sox17:GFP*))	Tg(*kdrl:mCherry*)
148	3-T-0129-1	female	diploid	yes	PG (Tg(*sox17:GFP*))	Tg(*kdrl:mCherry*)
149	3-T-0129-2	female	diploid	yes	PG (Tg(*sox17:GFP*))	Tg(*kdrl:mCherry*)
150	3-T-0129-3	male	diploid	yes	PG (Tg(*sox17:GFP*))	Tg(*kdrl:mCherry*)
151	3-T-0129-4	female	diploid	yes	PG (Tg(*sox17:GFP*))	Tg(*kdrl:mCherry*)
152	3-T-0129-5	female	diploid	yes	PG (Tg(*sox17:GFP*))	Tg(*kdrl:mCherry*)
153	3-T-0129-6	male	diploid	yes	PG (Tg(*sox17:GFP*))	Tg(*kdrl:mCherry*)
154	3-T-0129-7	male	diploid	yes	PG (Tg(*sox17:GFP*))	Tg(*kdrl:mCherry*)
155	3-T-0130-1	female	diploid	yes	PG (Tg(*sox17:GFP*))	Tg(*kdrl:mCherry*)
156	3-T-0130-2	male	diploid	yes	PG (Tg(*sox17:GFP*))	Tg(*kdrl:mCherry*)
157	3-T-0130-4	male	diploid	yes	PG (Tg(*sox17:GFP*))	Tg(*kdrl:mCherry*)
158	3-T-0130-5	female	diploid	yes	PG (Tg(*sox17:GFP*))	Tg(*kdrl:mCherry*)
159	3-T-0130-6	male	diploid	yes	PG (Tg(*sox17:GFP*))	Tg(*kdrl:mCherry*)
160	3-G1S-0129	female	diploid	yes	PG (Tg(*sox17:GFP*))	Tg(*kdrl:mCherry*)
161	4-T-0209-1	male	diploid	yes	PG (Tg(*sox17:GFP*))	Tg(*kdrl:mCherry*)
162	4-T-0209-2	male	diploid	yes	PG (Tg(*sox17:GFP*))	Tg(*kdrl:mCherry*)
163	4-T-0209-3	male	diploid	yes	PG (Tg(*sox17:GFP*))	Tg(*kdrl:mCherry*)
164	4-T-0209-4	male	diploid	yes	PG (Tg(*sox17:GFP*))	Tg(*kdrl:mCherry*)
165	4-G1S-0209	male	diploid	yes	PG (Tg(*sox17:GFP*))	Tg(*kdrl:mCherry*)

ND: not detected

Table S4. Sex ratio of semi-cloned zebrafish produced in this study

Donor Cell Line	Recipient egg	Sex		female/male
		female	male	
Tg(*sox17:GFP*)-AG	AB	1	0	
Tg(*fli1a:EGFP*)-AG	Tg(*kdrl:mCherry*)	1	0	
Tg(*fli1a:EGFP*)-PG	Tg(*kdrl:mCherry*)	1	1	1
Tg(*kdrl:mCherry*)-PG	Tg(*sox17:GFP*)	8	6	1.33
Tg(*sox17:GFP*)-PG	Tg(*kdrl:mCherry*)	69	32	2.16
Tg(*sox17:GFP*)-PG	AB	30	13	2.31
subtotal		110	52	2.12

Table S5. Primers used in PCR analysis and genotyping

Category	Gene name	Forward Primer (5'-3')	Reverse Primer (5'-3')
genotyping	*GFP*	ACGTAACGGCCACAAGTTC	CTGGGTGCTCAGGTAGTGGT
	mCherry	ATGAAGGGCGAGGAGGATAACA	CTTGTACAGCTCGTCCATGCC
knock-out	*tbxta*	CTTGAAGACGCGGAGTTGTG	GCTTTGGGGTTCGGGTTTC
	smo	GCACCGCAATTGTACGATGA	AGCCAGAATCAGAGACGTGT
	tfap2a	CATTTGGTTGGCGCTGTACT	TTGCATGAAGACCTGACAGC
	capn3a	AGCAGCCCAAAGAGAAGACT	TCCCATGAGCCATTTCAACA
	capn8	CCCCTGCTCCTATTCGCTTA	TGGACTCTGAGCAAACAAATGG
	capn12	AGGGATCTGGTGTGTTAGGC	GCATGTTAAGCTGGTTTGAGAC
knock-in	*huc*	AGACTTGCTTTGTTTACCAAATA AGTGGATTTGGATTTGC	CATGAACTCCTTGATGATGGCCA TGTTATCCTCCTCGCCC
NT-SC	*capn12*	GTTTGGTGTCGTTCCTGAGG	GTGGCCGGTGAGTGTAGATT

Table S6. sgRNA target sites used in the study.

Category	Target Site	Location	Target Sequence (PAM)	Reference
knock-out	*tbxta*	exon 2	CGACCCTAATGCAATGTACT(CGG)	[23, 24]
	smo	exon 1	GGGGCGCTTGGAGGACATCT(TGG)	[23, 24]
	tfap2a	exon 4	CTGAACGCTTCCTTGCTCGG(CGG)	[23, 24]
	capn3a	exon 1	AGATGCCCTATACGCCGTCT(GGG)	
	capn8	exon 1	GGACTACCAAAGCCTGAAGC(AGG)	
	capn12	exon 3	TGCCAGCGTAGGATTCACTC(AGG)	
knock-in	*huc*	intron 7	GATATGATAGTTGCTCGTGG(AGG)	
NT-SC	*capn12-1*	intron 3	TGGCTGCACTCATTTGTCAA(TGG)	
	capn12-2	intron 4	AGTGGTAACACTGTAATTTA(AGG)	

Table S7. Comparation of three semi-cloned strategies in this study.

	donor embryo pre-screen	donor cell preservation	Gene edit types	genotype of semi-cloned zebrafish	Need HCNT (haploid cell nuclear transfer) ?	Operation complexity	Number of RNP injected embryos needed	Number of NT embryos needed
**Strategy A (Fig. S7a)**	no	fresh	KO	various	no	easy	50	0
**Strategy B (Fig. S9b)**	yes	4℃ 4h	KI	various	no	easy	200	0
**Strategy C (Fig. 1i)**	yes	4℃ overnight	KO, KI	defined	yes	difficult	50	20

Supplementary Videos

Video S1. Intracytoplasmic haploid blastula cell injection using pneumatic operation system

Video S2. Intracytoplasmic haploid blastula cell injection using piezo system

Video S3. Mitotic progression of synchronized cells after release

References

15 Xia, Z. *et al. Dev Biol* **381**, 83-96, doi:10.1016/j.ydbio.2013.06.009 (2013).

16 Lawson, N. D. & Weinstein, B. M. *Dev Biol* **248**, 307-318, doi:10.1006/dbio.2002.0711 (2002).

17 Chung, W. S. & Stainier, D. Y. *Dev Cell* **14**, 582-593, doi:10.1016/j.devcel.2008.02.012 (2008).

18 Rezaei, M. *et al. Galen Med J* **8**, e1068, doi:10.31661/gmj.v8i0.1068 (2019).

19 Pauls, S., Geldmacher-Voss, B. & Campos-Ortega, J. A. *Dev Genes Evol* **211**, 603-610, doi:10.1007/s00427-001-0196-x (2001).

20 Kimmel, C. B., Ballard, W. W., Kimmel, S. R., Ullmann, B. & Schilling, T. F. *Dev Dyn* **203**, 253-310, doi:10.1002/aja.1002030302 (1995).

21 Kroeger, P. T., Jr. *et al. J Vis Exp*, doi:10.3791/51708 (2014).

22 Endoh, M., Fujimoto, T., Yamaha, E. & Arai, K. *Zebrafish* **15**, 33-44, doi:10.1089/zeb.2017.1482 (2018).

23 Wu, R. S. *et al. Dev Cell* **46**, 112-125 e114, doi:10.1016/j.devcel.2018.06.003 (2018).

24 Hoshijima, K. *et al. Dev Cell* **51**, 645-657 e644, doi:10.1016/j.devcel.2019.10.004 (2019).
